# Evaluating Human T-Cell Therapy of Cytomegalovirus Organ Disease in HLA-Transgenic Mice

**DOI:** 10.1371/journal.ppat.1005049

**Published:** 2015-07-16

**Authors:** Simone Thomas, Sebastian Klobuch, Jürgen Podlech, Bodo Plachter, Petra Hoffmann, Angelique Renzaho, Matthias Theobald, Matthias J. Reddehase, Wolfgang Herr, Niels A. W. Lemmermann

**Affiliations:** 1 Department of Internal Medicine III, Hematology and Oncology, University Hospital of Regensburg, Regensburg, Germany; 2 Regensburg Center of Interventional Immunology, University of Regensburg, Regensburg, Germany; 3 Department of Internal Medicine III, Hematology, Oncology and Pneumology, University Medical Center of the Johannes Gutenberg-University, Mainz, Germany; 4 Institute for Virology and Research Center for Immunotherapy (FZI), University Medical Center of the Johannes Gutenberg-University, Mainz, Germany; Oregon Health & Science University, UNITED STATES

## Abstract

Reactivation of human cytomegalovirus (HCMV) can cause severe disease in recipients of hematopoietic stem cell transplantation. Although preclinical research in murine models as well as clinical trials have provided 'proof of concept' for infection control by pre-emptive CD8 T-cell immunotherapy, there exists no predictive model to experimentally evaluate parameters that determine antiviral efficacy of human T cells in terms of virus control in functional organs, prevention of organ disease, and host survival benefit. We here introduce a novel mouse model for testing HCMV epitope-specific human T cells. The HCMV UL83/pp65-derived NLV-peptide was presented by transgenic HLA-A2.1 in the context of a lethal infection of NOD/SCID/IL-2rg^-/-^ mice with a chimeric murine CMV, mCMV-NLV. Scenarios of HCMV-seropositive and -seronegative human T-cell donors were modeled by testing peptide-restimulated and T-cell receptor-transduced human T cells, respectively. Upon transfer, the T cells infiltrated host tissues in an epitope-specific manner, confining the infection to nodular inflammatory foci. This resulted in a significant reduction of viral load, diminished organ pathology, and prolonged survival. The model has thus proven its potential for a preclinical testing of the protective antiviral efficacy of HCMV epitope-specific human T cells in the evaluation of new approaches to an immunotherapy of CMV disease.

## Author Summary

Pre-emptive CD8 T-cell therapy of human cytomegalovirus (HCMV) disease in immunocompromised recipients of hematopoietic stem cell transplantation gave promising results in clinical trials, but limited efficacy and the need of HCMV-seropositive memory cell donors has so far prevented adoptive cell transfer from becoming clinical routine. Further development is currently hampered by the lack of experimental animal models that allow preclinical testing of the protective efficacy of human T cells in functional organs. While humanized mouse models with human tissue implants are technically and statistically demanding, and are limited to studying human T-cell activation and local virus control in the implants, a more feasible model for control of systemic infection and prevention of multiple-organ CMV disease is regrettably missing. Here we introduce such a model based on infection of genetically immunocompromised, HLA-A2.1-transgenic NOD/SCID/IL-2rg^-/-^ mice with a chimeric murine CMV engineered to express the HCMV NLV-peptide epitope. Mimicking the scenario of HCMV-unexperienced donors, human T cells transduced with a human T-cell receptor specific for HLA-A.2.1-presented NLV peptide controlled systemic infection and moderated organ disease resulting in a survival benefit. The model promises to become instrumental in defining T-cell properties that determine their protective efficacy for a further development of adoptive immunotherapy of post-transplantation CMV infection.

## Introduction

Reactivation of latent human cytomegalovirus (HCMV) infection is a frequent complication in patients after allogeneic hematopoietic stem cell transplantation (HSCT). Although potent antiviral drugs are available, their usage, however, is often limited by hematotoxicity and nephrotoxicity. In addition, the broad application of these drugs during pre-emptive treatment strategies is associated with a higher frequency of late-onset HCMV disease [1,2]. Preclinical research in murine models ([3–6], reviewed in [7–9]) as well as clinical phase I/II trials ([10–12], reviewed in [13,14]) have shown that the adoptive transfer of virus-specific CD8 T cells is a promising therapeutic option for preventing and treating CMV disease. However, the feasibility of HCMV-specific immunotherapy is currently impeded in clinical routine due to technical restrictions. It has also limitations in case the donor is HCMV-seronegative or carries only low frequencies of HCMV-specific memory T cells. In this situation, transduction of non-cognate T cells with virus specific T-cell receptors (TCR) may be an alternative means to transfer HCMV-specific T-cell function into HSCT recipients [15,16]. In any case, clinical protocols need to be improved before HCMV-specific cell therapy can be implemented in general clinical practice. To allow for a more reliable analysis of HCMV immunotherapies (e.g. adoptive T-cell therapy, therapeutic vaccination) animal models that mimic HCMV infections are needed.

Since HCMV replication is strictly restricted to cells and tissues of human origin ([17], reviewed in [18]), previous animal models utilized murine CMV (mCMV) as surrogate virus (reviewed in [7–9]) or mice infected with HCMV after implantation with human cells or tissues, for instance with tumor cell lines, fetal thymus, and liver biopsies ([19–23], reviewed in [24]). The implantation approach has shown that HCMV strains replicate locally with differences in pathogenicity, but fail to spread between tissue implants. To support systemic infection, Smith et al. [25] infected human CD34^+^ hematopoietic stem cell-engrafted mice with HCMV to establish latency and to induce virus reactivation in tissue-migrated monocytes and macrophages by granulocyte-colony stimulating factor (G-CSF) treatment. By model design, however, viral dissemination to functional organs relevant for viral pathogenesis (e.g. spleen, lungs, and liver) and transmission (salivary glands) cannot be assessed even in these advanced humanized mouse models.

We herein present a novel preclinical mouse model that allows the direct testing of HCMV-specific human T-cell products. In this, we combined the well-described murine model of mCMV infection of the immunocompromised host (reviewed in [7–9]) with the strong T-cell immunogenicity of the HLA-A*0201 (HLA-A2.1) restricted HCMV epitope pp65_495-503_ NLVPMVATV (briefly, NLV) [26]. We generated a chimeric recombinant mCMV expressing the NLV epitope (mCMV-NLV) during the infectious cycle to allow organ manifestation of the infection in the natural host similar to that seen in immunocompromised patients. After infection of HLA-A2.1 transgenic, constitutively combined-immunodeficient NOD/SCID/IL-2rg^-/-^ (NSG/HHD) mice, which lack cells of adaptive immunity and are additionally deficient in natural killer (NK) cells [27], mCMV-NLV resulted in a rapid systemic infection that could be effectively combated by adoptively transferred human NLV-specific CD8 T cells as well as by human T cells transduced with an NLV-specific TCR. After migration into murine organs, these T cells not only reduced mCMV-NLV titers in an epitope-specific manner but also prolonged survival of infected mice, despite continued combined immunodeficiency in absence of hematopoietic reconstitution.

For the clinical correlate of reactivated HCMV infection associated with HSCT, our findings predict that pre-emptive CD8 T-cell immunotherapy, even though controlling CMV only transiently, can bridge the critical time of an immunocompromised state until immune reconstitution by HSCT takes over. Altogether, we provide a novel animal infection model that allows the direct evaluation and preclinical testing of HCMV-specific human T cells and potentially other HCMV immunotherapy approaches, including vaccination of HSCT recipients in parallel to hematopoietic reconstitution.

## Results

### Integration of the HCMV-NLV epitope into mCMV protein IE2 does not alter mCMV growth

**Fig 1:**
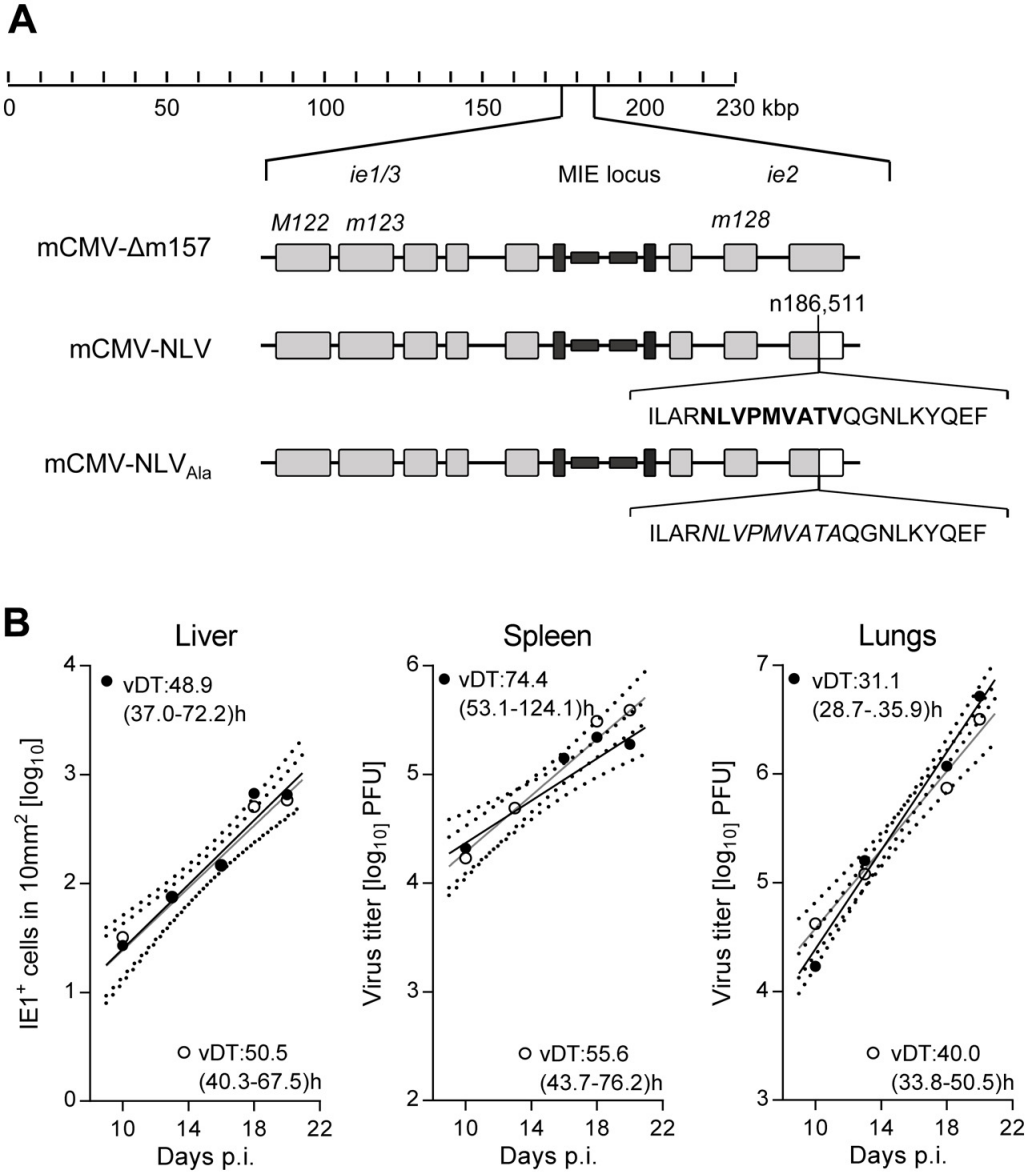
Construction and characterization of recombinant mCMV-NLV expressing the HLA-A2.1 restricted peptide NLV. (A) Map of the major immediate-early (MIE) locus of mCMV, illustrating the integration of the sequence of the immunodominant HCMV peptide derived from protein pUL83/pp65 (bold face capital letters) or of the loss-of-function Ala variant (capital letters in italics) within mCMV ORF*m128*. Both peptide sequences were flanked by their authentic amino acid residues present in pUL83/pp65. Grey-shaded boxes represent exons, black bars and boxes symbolize the bidirectional promoters and enhancers, respectively. (B) Viral growth curves *in vivo* represented by the time course of numbers of infected IE1^+^ liver cells in representative 10-mm^2^ areas of liver tissue sections (left panel) or of virus titers in homogenates of spleen (center panel) and lungs (right panel) after intraplantar infection of NSG/HHD mice with 1x10^5^ PFU of mCMV-Δm157 (filled circles) or mCMV-NLV (open circles). Symbols represent median values from 4 to 5 mice per group and time of assay. Log-linear regression lines (based on data from all individual mice) and their corresponding 95% confidence areas (bordered by dotted curves) are indicated. Viral doubling times (vDT) and their 95% confidence intervals (in parentheses) are given.

The key strategy of our model was to antigenically ‘humanize’ both the murine virus and the murine host tissue for HLA class I-restricted recognition of infected cells by human T cells in functional host organs. For construction of the chimeric virus mCMV-NLV, we integrated the coding gene sequence of the HLA-A2.1-restricted pp65 (UL83)_495-503_ peptide epitope NLVPMVATV (abbreviated: NLV) of HCMV, with its natural flanking amino acids for authentic proteasomal cleavage and precursor peptide sequence, into the immediate-early (IE)2/m128 gene of mCMV-Δm157 (Fig 1A). Although NSG/HHD mice are deficient in NK cell activity due to lack of the interleukin-2 receptor (IL-2R) common γ chain (CD132) causing deficient IL-2/4/7/9/15/21 signaling [28,29], mCMV-Δm157 was chosen as parental virus to also formally exclude activation of the Ly49H^+^ subset of murine NK cells by m157-Ly49H interaction, a feature that is only valid in the C57BL/6 genetic background and might thus interfere with a broader application of the model (for a review, see [30]). For control experiments, a lack-of-epitope mCMV-NLV derivative was generated, in which a single amino acid replacement at the C-terminal HLA-A2.1 anchor position valine (V_503_) by alanine (mCMV-NLV_Ala_) prevents epitope generation in the proteasome and MHC/HLA class-I (MHC/HLA-I) presentation (Fig 1A) (for the concept, see [31,32]).

The IE2/m128 gene encodes a regulatory but non-essential protein expressed in the IE phase of viral replication [33,34] and sporadically also during viral latency [35]. To verify, in the first place, that integration of the NLV epitope into the IE2 protein does not attenuate the virus, which otherwise would render the model inapplicable for studying viral pathogenesis, we compared the *in vivo* growth kinetics of parental virus mCMV-Δm157 and its antigenicity variant mCMV-NLV in NSG/HHD mice. In selected organs relevant for viral pathogenesis, such as liver, spleen, and lungs (Fig 1B), the two viruses were found to replicate in a log-linear fashion with comparable doubling times within each organ (for the principle, see [36,37]), though with organ-typic numerical differences, with most aggressive growth in the lungs. In conclusion, insertion of the NLV epitope did not impair *in vivo* viral growth. The chimeric virus thus fulfills the model’s first prerequisite of an unaltered replicative fitness.

### The HCMV-NLV epitope is successfully processed and presented by HLA-A2.1 transgenic murine cells

**Fig 2:**
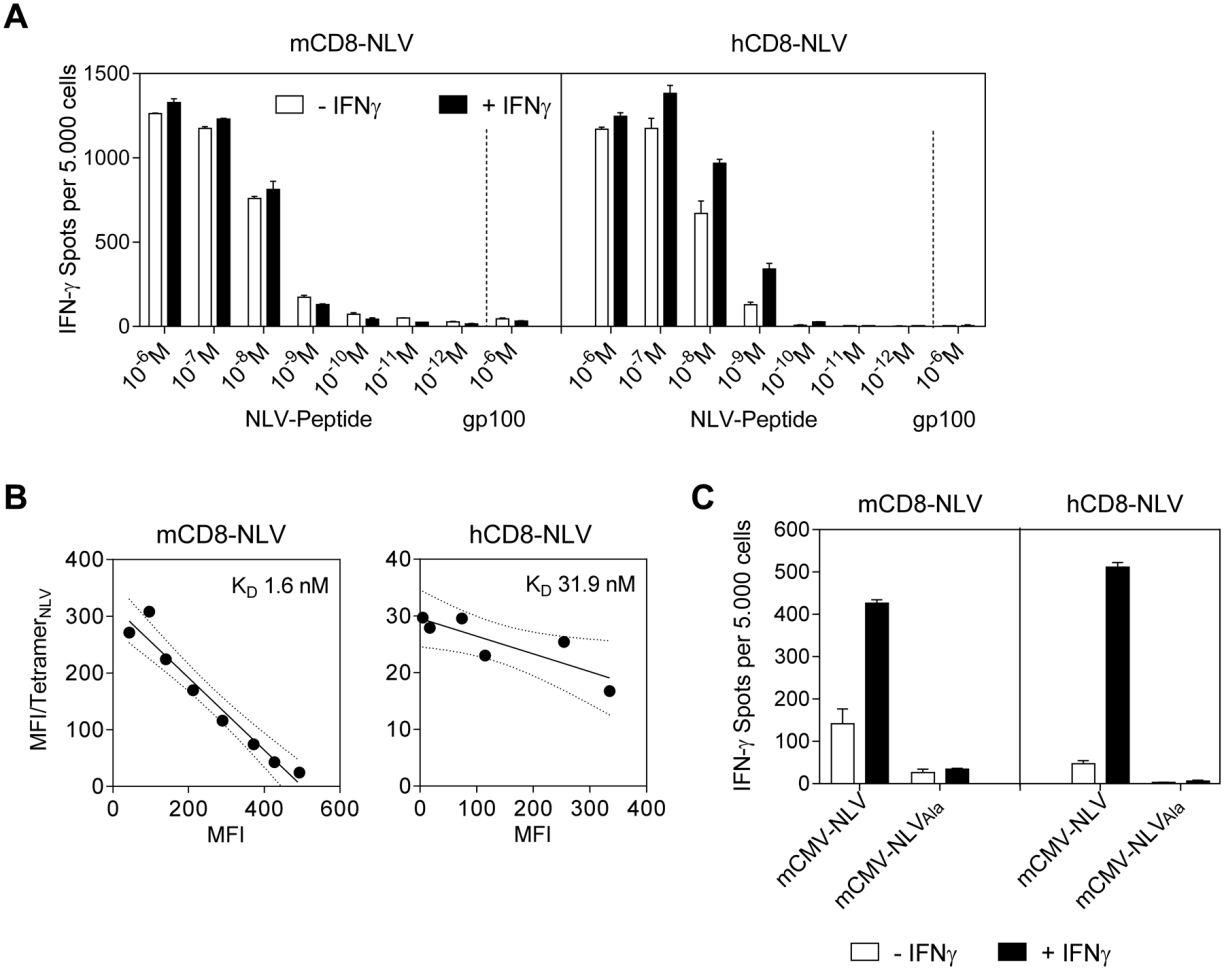
Epitope recognition by NLV peptide specific CD8 T cells. (A) IFN-γ spots, each representing a CD8 T cell responding with IFN-γ production, formed upon sensitization of NLV peptide-specific murine (mCD8-NLV; left panel) and human (hCD8-NLV; right panel) cytolytic CD8 T-cell lines (CTLL) by NLV-peptide-pulsed NSG/HHD MEF at an effector-to-stimulator cell ratio of 0.1:1. MEF were pretreated with murine IFN-γ for 48h (filled bars) or left untreated (open bars) and were exogenously loaded with synthetic NLV- or non-cognate gp100_280-288_-peptide at the indicated concentrations. Data are shown as mean of duplicates from one experiment representative of two performed. Error bars represent the range. (B) Structural avidity of antigen binding to mCD8-NLV and hCD8-NLV CTLL was quantified by dose-dependent HLA-A2.1/NLV tetramer binding in flow cytometry. The respective dissociation constant (K_D_) was calculated from half-maximal tetramer binding obtained by Scatchard plot analysis. Dotted curves border the 95% confidence regions of the log-linear regression lines. MFI, mean fluorescence intensity. (C) HLA-A2.1 restricted presentation of NLV epitope by NSG/HHD MEF pretreated with murine IFN-γ for 48h (filled bars) or left untreated (open bars) and infected at an MOI of 4 with the indicated viruses for a total time of 22h until the end of the assay. Peptide presentation on the infected MEF during that period was detected in an IFN-γ ELISpot assay with mCD8-NLV (left panel) and hCD8-NLV (right panel) CTLL at an effector-to-stimulator cell ratio of 0.1:1. Data are shown as mean of duplicates from one experiment representative of two performed. Error bars represent the range.

The second prerequisite of the model is that the NLV peptide is actually generated by processing of the chimeric IE2-NLV protein in mCMV-NLV infected cells and that it is presented in association with HLA-A2.1 at the cell surface for recognition by TCR. To directly assess presentation, an endpoint which includes the preceding processing events, we chose two independent NLV peptide-specific, cytolytic CD8 T cell lines (CTLL) as probes: (i) a published, peptide-selected murine long-term CTLL, mCD8-NLV, expressing a murine TCR [38] for serving as a murine reference line, and (ii) a freshly generated peptide-selected human short-term polyclonal CTLL, hCD8-NLV, expressing human TCRs for modeling the situation of an HCMV-experienced, seropositive cell transfer donor as the clinical correlate. While mCD8-NLV consisted of cells with effector phenotype (CD44^+^CD62L^low^ T_E/EM_), including effector-memory cells, early effector cells, and short-lived effector cells ([39,40], reviewed in [41,42]) the short-term hCD8-NLV line was composed of cells with central memory and effector memory phenotype, T_CM_ and T_EM_, respectively (S1 Fig). The functional avidities of these two CTLL were then tested in an IFN-γ secretion-based ELISpot assay for their sensitization by HLA-A2.1 transgenic NSG/HHD mouse embryonic fibroblasts (HLA-A2.1-MEF) exogenously loaded with graded concentrations of synthetic NLV peptide (Fig 2A). Notably, when corrected for background response measured with an unrelated peptide, both CTLL were similarly sensitive with an endpoint NLV peptide concentration of 10^−9^ M. Pretreatment of the HLA-A2.1-MEF stimulator cells with IFN-γ, which is known to enhance MHC class-I expression [43], only modestly increased the numbers of CTLL cells responding to exogenously peptide-loaded HLA-A2.1 molecules. Contrasting with the comparable functional avidities of the two CTLL, an assay based on their physical capacity to bind NLV peptide-folded HLA-A2.1 tetramers [44,45] revealed a higher structural avidity of mCD8-NLV (Fig 2B), which predicts more efficient *in vivo* function [8,46]. After this basal characterization of the two CTLL, we tested their capacity to detect naturally processed NLV peptide presented by HLA-A2.1 on the surface of infected NSG/HHD HLA-A2.1-MEF (Fig 2C). In both lines, a significant, though low, number of cells had an avidity sufficient for recognizing NLV-HLA-A2.1 complexes presented on infected cells. This number, however, was significantly enhanced when NSG/HHD HLA-A2.1-MEF were treated with IFN-γ prior to infection, which contrasts to the only modest effect of IFN-γ on exogenous peptide loading. The explanation likely is the overriding of the function of viral immune evasion molecules (viral regulators of antigen presentation, vRAPs) by IFN-γ, as described previously to occur in infected cells *in vitro* as well as in infected tissues *in vivo* ([47,48], reviewed in [49]).

In this context it is worth noting that the immune evasion molecules of mCMV indeed also target the transgenic HLA-A2.1 (S2 Fig). Specifically, as shown by flow cytometry, immune evasion mechanisms of mCMV caused cell surface down-modulation of both murine H2-K^d^ and the transgenic human HLA-A2.1 molecules in infected (gp36.5/m164^+^) but not in uninfected (gp36.5/m164^-^) cells of the same infected cell cultures when compared to uninfected control cultures. Accordingly, H2-K^d^ and HLA-A2.1 expression levels remained largely unaffected upon infection with the immunoevasin gene deletion mutant mCMV-ΔvRAP (S2 Fig) [48,50,51].

Importantly, both the normal and the IFN-γ-enhanced recognition of infected cells proved to be strictly NLV epitope-specific, as shown by baseline values of response to NSG/HHD HLA-A2.1-MEF infected with virus mCMV-NLV_Ala_ (Fig 2C).

### NLV peptide-specific human CD8 T cells are protective by confining the infection of HLA-A2.1 transgenic murine tissues to nodular inflammatory foci (NIF) in an epitope-specific manner

After fulfillment of the model’s prerequisites of unaltered virulence and of successful transgenic antigen presentation, both NLV peptide-specific CTLL, characterized as detailed above, were tested for their capacity to control the replication of chimeric virus mCMV-NLV in tissues of HLA-A2.1 transgenic NSG/HHD mice, which can be engrafted with components of the human immune system due to a lack of an adaptive immune system and innate immune defects [27]. Since NSG/HHD mice are devoid of endogenous murine T cells, tissue infiltration by T cells and antiviral control can be attributed exclusively to the transferred cells.

**Fig 3:**
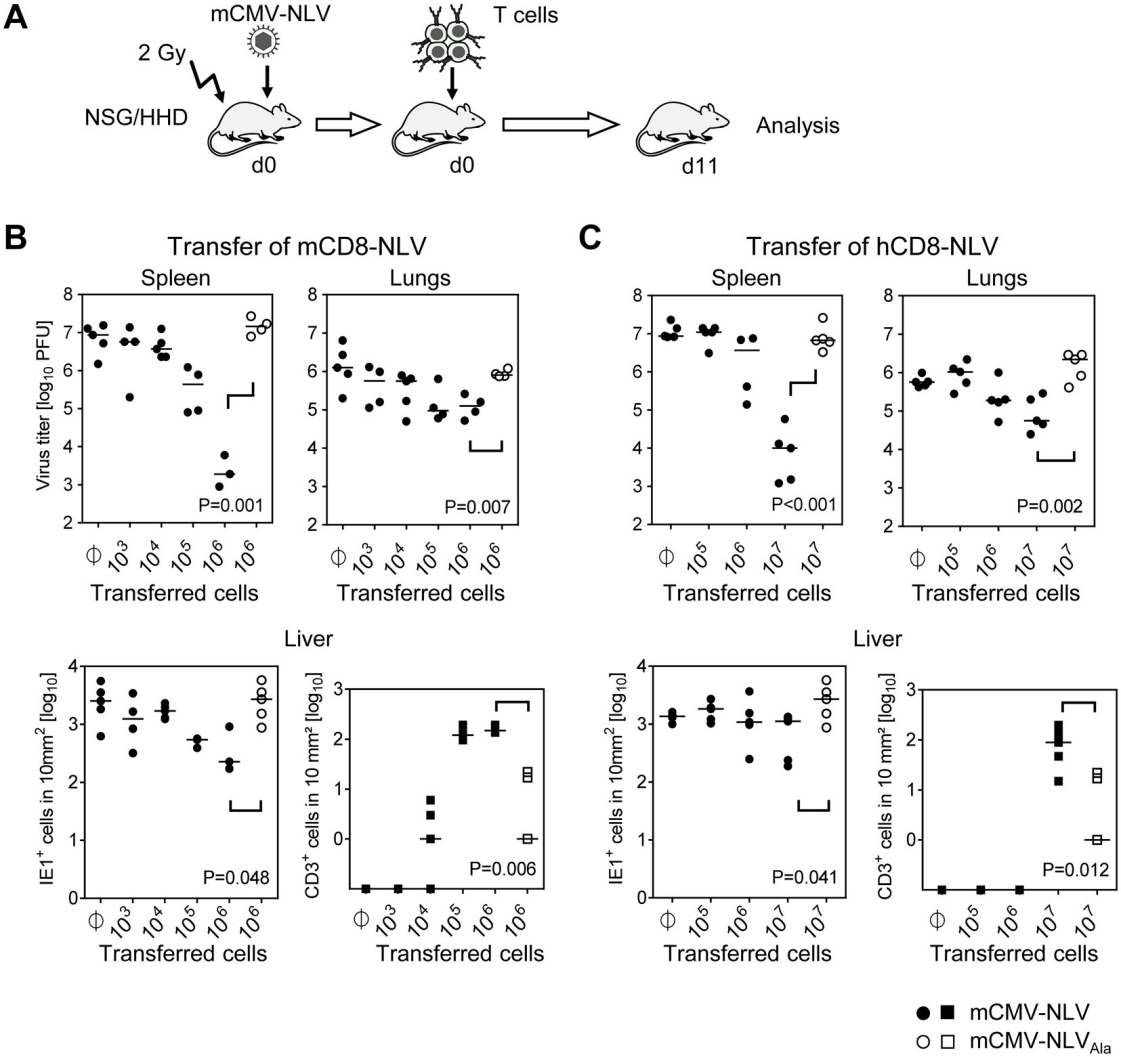
Dose-dependent antiviral effect of NLV-peptide-specific CD8 T cells in mCMV-NLV infected NSG/HHD mice. (A) Experimental strategy and schedule of adoptive transfer of NLV-specific CD8 T cells into infected NSG/HHD recipients that were preconditioned by total-body γ-irradiation with a dose of 2 Gy. Intraplantar infection was performed throughout with 1x10^5^ PFU of chimeric mCMV. Recipients (n = 4–5 per group) were infected with mCMV-NLV (filled symbols) or mCMV-NLV_Ala_ (open symbols), followed by i.v. transfer of graded numbers of (B) murine or (C) human NLV-specific CD8 T cells (mCD8-NLV or hCD8-NLV CTLL, respectively). Infectivity was quantified at d11 post-transfer in spleen and lungs by standard plaque (plaque-forming unit, PFU, assay). In the livers of the same mice, infected cells (filled and open circles for mCMV-NLV and mCMV-NLV_Ala_, respectively) and tissue-infiltrating CD8 T cells (filled and open squares, correspondingly) were stained by 2C-IHC and counted in representative 10-mm^2^ areas of tissue sections. Symbols represent individual mice and horizontal bars mark median values. Statistical analysis for group differences of most interest (bracketed) was performed after log-transformation using Student’s t-test (unpaired, two-sided; p<0.05 considered significant) with Welch’s correction.

Specifically, sublethally (2 Gy) γ-irradiated NSG/HHD mice were infected by intraplantar injection with 1x10^5^ PFU of mCMV-NLV, a dose that would be lethal after approximately 12 to 17 days. Shortly thereafter, NLV-specific CTLL were intravenously transferred in order to mimic the clinical setting of pre-emptive therapy, and viral titers in spleen and lungs as well as numbers of infected cells in livers of individual mice were determined on day 11 (for the protocol scheme, see Fig 3A). In the case of transfer of human NLV-specific CTLL, single doses of IL-2 and IL-7 were given along with the cells for a short-term growth factor supply. In the beginning of the project, a key concern had been that a cross-species adoptive transfer of CD8 T cells might fail because of xenogeneic constraints in the chemokine-driven extravasion and tissue infiltration of intravenously transferred CD8 T cells. Not unexpectedly, based on extended literature to adoptive immunotherapy by CD8 T cells in fully murine models (reviewed in [7–9]), transfer of mCD8-NLV cells controlled the infection of spleen, lungs, and liver in a dose-dependent and epitope-specific fashion (Fig 3B). As shown for the liver, dose-dependent antiviral protection correlated with dose-dependent tissue infiltration by the adoptively transferred CD8 T cells. Likewise, lack of epitope after infection with mCMV-NLV_Ala_ abolished both tissue infiltration and control of virus replication. Importantly, as a new information, the results were qualitatively identical for the human CD8 T cells, hCD8-NLV, although higher cell numbers were needed (Fig 3C). Whether this relates to the lower structural avidity (recall Fig 2B) or to xenogeneic differences remains to be investigated. The more relevant conclusion, however, is that the functionality of antiviral human CD8 T cells can, in principle, be studied in murine tissues.

**Fig 4:**
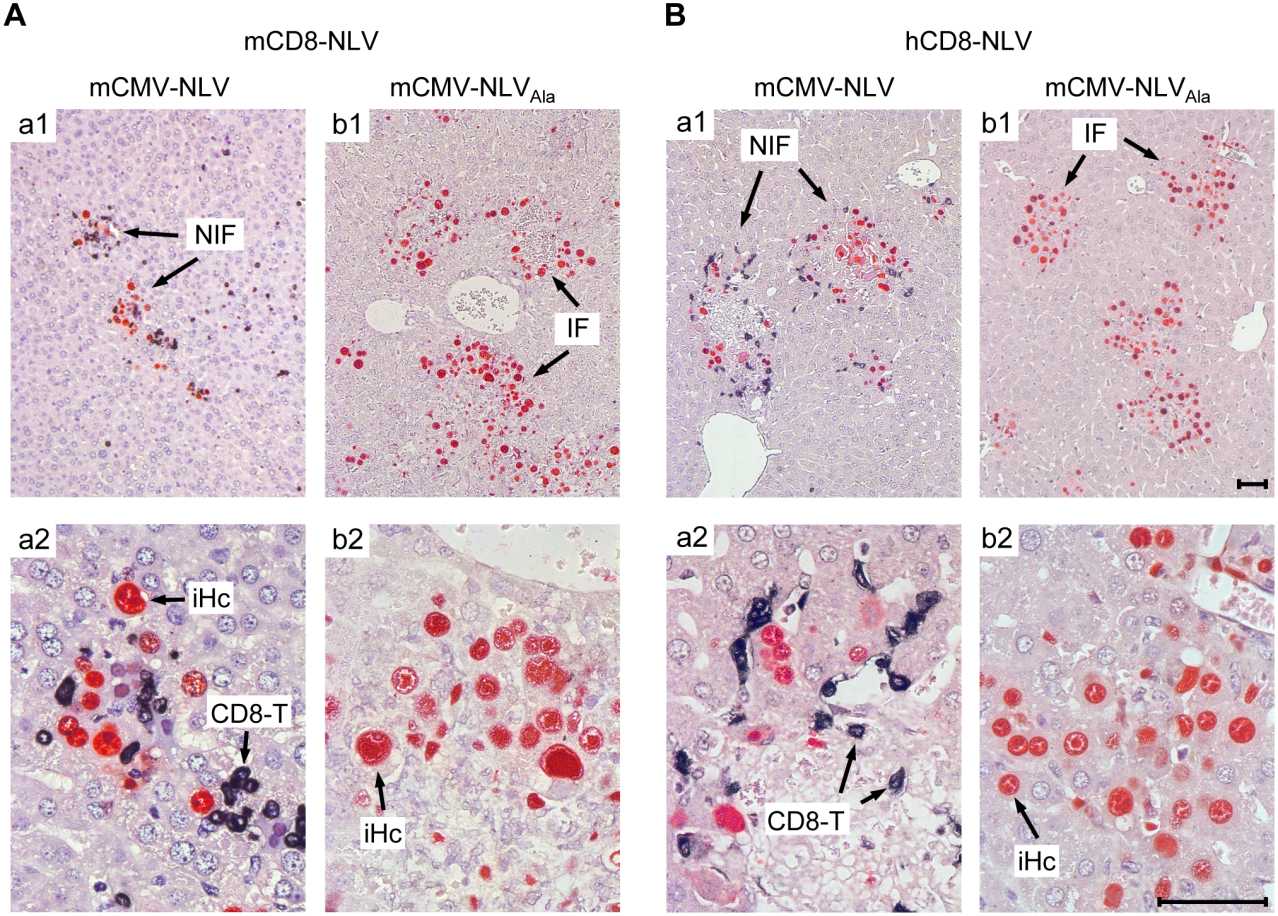
Protective NIF formation after adoptive transfer of NLV-specific murine and human CD8 T cells. Corresponding to Fig 3, 2C-IHC of liver tissue sections taken on d11 after transfer of (A) mCD8-NLV CTLL or (B) hCD8-NLV CTLL show mCMV-NLV or mCMV-NLV_Ala_ infected hepatocytes (iHc, red staining of intranuclear IE1 protein) with typical intranuclear inclusion bodies, and infiltrating CD8 T cells (CD8-T, black cytoplasmic and membrane staining of CD3ε), forming nodular inflammatory foci (NIF) or foci of infection in absence of CD8 T cells (IF), respectively. Upper row images give overviews (A,B; a1, b1), higher-magnification lower row images (A, B; a2, b2) reveal details. Bar markers represent 50 μm throughout.

Protection against disseminated CMV organ infection has a microanatomical correlate in the formation of nodular inflammatory foci (NIF) [52–57]. As shown previously in a related murine model [54] and reproduced here for the example of mCD8-NLV cells, infection by a virus expressing the cognate epitope is confined to NIF where control takes place and where infected tissue cells and infiltrating CD8 T cells co-localize. In contrast, in the absence of the cognate epitope [54] after infection with virus mCMV-NLV_Ala_, CD8 T cells do not infiltrate infected tissue at all, can thus not form NIF, and, accordingly, allow more widespread infection visible as many foci of infection (IF), with the consequence of virally-caused tissue pathology (Fig 4A). Importantly, these rules were found to apply, in principle, also to the human CD8 T cells, hCD8-NLV, although NIF appeared as being somewhat less condensed (Fig 4B). This may relate to the lower structural avidity (recall Fig 2B) and the higher cell numbers needed for antiviral protection (recall Fig 3C). Again, although the syngeneic cell transfer system is more efficient, which is not surprising, a xenogeneic system also allows NIF formation and control of intra-tissue virus spread, despite the species barrier.

### TCR-transduced human CD8 T cells control the infection of HLA-A2.1 transgenic NSG/HHD mice

The need of an HCMV-experienced (HCMV antibody positive) donor as a source for virus-specific memory CD8 T cells is a limitation for an adoptive immunotherapy of HCMV reactivation in the highest-risk constellation of an HCMV-seronegative donor and—seropositive recipient (D^-^R^+^) [58–61], which can currently only be overcome by an HLA-matched third party memory T-cell donor [12,62,63]. An alternative approach, already successfully applied in tumor models [64,65], is the transduction of T cells derived from the HLA-matched HSCT donor with a virus-epitope specific TCR.

**Fig 5:**
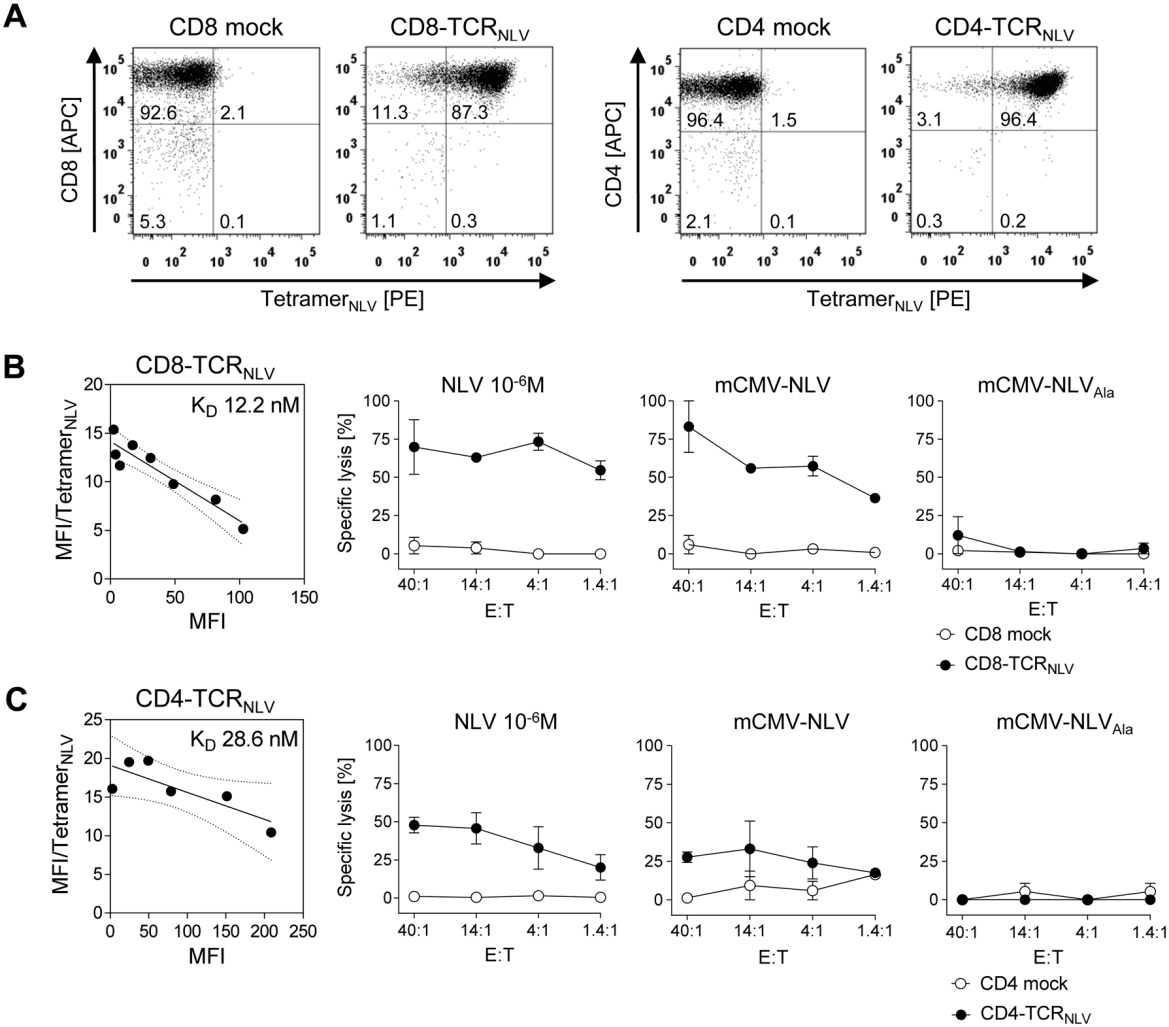
Transgenic TCR_NLV_ expression, structural avidity, and cytolytic activity of TCR-transduced human T-cell subsets. (A) Immunomagnetically selected human CD8 T cells (left panels) and CD4 T cells (right panels) were retrovirally transduced with TCR_NLV_ (CD8-TCR_NLV_ and CD4-TCR_NLV_, respectively) or empty vector (CD8 mock and CD4 mock, respectively) and were drug-selected. After *in vitro* expansion with anti-CD3/CD28 beads for a period of 10d, cells were analyzed cytofluorometrically for expression of CD4 and CD8, as well as of TCR_NLV_ using HLA-A2.1/NLV tetramers. Shown are 2D dot plots with the percentages of labeled cells indicated. (B) and (C) Characterization of CD8-TCR_NLV_ and CD4-TCR_NLV_ cells respectively. (Left panel) Structural avidity (K_D_) of antigen binding was quantified by dose-dependent HLA-A2.1/NLV tetramer binding determined by flow cytometry (see the legend of Fig 2B). (Right panels) Cytolytic activities of TCR_NLV_-transduced and mock-transduced T cells (filled and open circles, respectively) determined by a standard [^51^Cr]-release assay at the indicated effector (i.e. T cell) to target (i.e. MEF of NSG/HHD mice) cell ratios (E:T). NSG/HHD MEF were pre-treated with IFN-γ for 48h and were either exogenously loaded with a high dose of synthetic NLV peptide as a positive control, or infected for 12h with mCMV-NLV and, as a non-antigenic control, with mCMV-NLV_Ala_. Data represent means of duplicate assay cultures. Error bars indicate the range.

To model this clinical need, a first application of the here newly described HLA-A2.1 transgenic mouse CMV infection model was to test the *in vivo* antiviral function of human CD8 as well as CD4 T cells that were transduced by retroviral gene transfer of a modified high-affinity human T-cell receptor α/β (TCR_NLV_) previously shown to reprogram CD8 and CD4 T cells derived from HCMV-seronegative donors [16]. As a basal characterization of the TCR_NLV_ transduced T cells enriched by drug selection, cytofluorometric analysis with NLV peptide-folded HLA-A2.1 tetramers for TCR_NLV_ staining shows high transduction efficacies (Fig 5A), and Fig 5B and 5C reveal structural avidity as well as NLV epitope-specific cytolytic potential of CD8-TCR_NLV_ and CD4-TCR_NLV_ cells, respectively. Using NSG/HHD HLA2.1-MEF exogenously loaded with a high dose of synthetic NLV peptide as a positive standard, both transduced T-cell lines specifically lysed IFN-γ pre-treated mCMV-NLV-infected NSG/HHD cells, overcoming the presence of the mCMV immune evasion molecules (see above, [48]), and this lysis was TCR/epitope-specific as indicated by its absence after mock-transduction of the T cells as well as after infection of the target cells with the antigenicity-loss variant mCMV-NLV_Ala_. Notably, CD8-TCR_NLV_ CTL, but not mock-transduced CD8 T cells, lysed HLA-A2.1-expressing human primary foreskin fibroblasts infected with the HCMV immune evasion gene (US2,3,6,11) deletion mutant RV-KB6 expressing the NLV peptide [66], but not those infected with the combined US2,3,6,11 and pp65/UL83 deletion mutant HCMV-RV-KB15 lacking NLV peptide [16] (S3 Fig). This, again, showed the specificity in terms of the need for the specific TCR and the cognate epitope presented by HLA-A2.1. For completing the basal characterization, S4A and S4B Fig show the phenotyping of the human CD8 and CD4 T cells, respectively, each before (upper row) and after (lower row) TCR_NLV_ transduction and subsequent lymphokine-driven expansion triggered by ligation of CD3 and CD28. In essence, and in accordance with previous studies on TCR transduction, the resulting CD8-TCR_NLV_ population consisted primarily of T_EM_ [67–70], whereas CD4-TCR_NLV_ cells were predominantly T_CM_.

**Fig 6:**
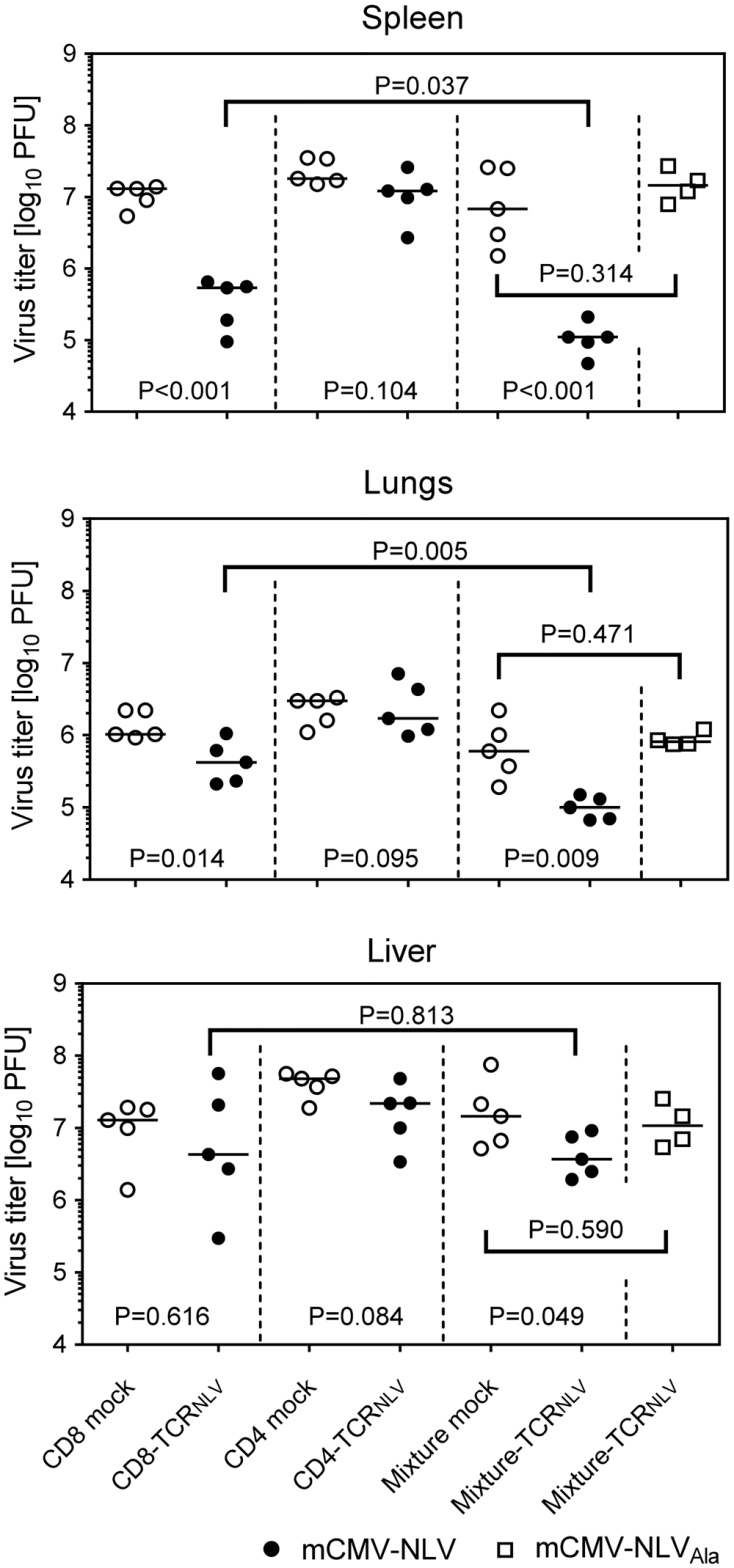
Antiviral effect of TCR_NLV_-transduced human CD4 and CD8 T cells upon adoptive transfer into mCMV-NLV infected NSG/HHD mice. Virus titers were determined in the indicated organs of mCMV-NLV-infected NSG/HHD mice adoptively transferred with 1x10^7^ mock-transfected (open circles) or TCR_NLV_-transfected (filled circles) CD8 or CD4 T cells or with 1:4 mixtures of CD4 (2x10^6^) and CD8 (8x10^6^) T cells (mixture mock: open circles; mixture-TCR_NLV_: filled circles). For controlling epitope specificity of the antiviral effect, virus titers were also determined in organs of mCMV-NLV_Ala_-infected mice adoptively transferred with the 1:4 mixture of TCR_NLV_-transduced CD4 and CD8 T cells (Mixture-TCR_NLV_; open squares). Organs were sampled for the virus plaque assay on d11 post-infection and adoptive T-cell transfer. For spleen and lungs, data refer to the whole organ; for the liver data are normalized to 0.5g of tissue. Symbols represent individual mice (n = 5 per group) and horizontal bars mark the median values. For statistical analysis, see the legend of Fig 2.

These cells, and the corresponding empty-vector transduction controls lacking cognate TCR, were then adoptively transferred into mCMV-NLV-infected NSG/HHD mice to test their *in vivo* antiviral efficacy (Fig 6). With the exception of the liver, where a tendential reduction in day 11 median virus titer did not reach statistical significance due to high variance in the therapy group, infectious virus load was found to be significantly reduced in spleen (P < 0.001) and lungs (P = 0.014) by 10^7^ CD8-TCR_NLV_ cells but not by 10^7^ CD4-TCR_NLV_ cells (P >0.05 throughout). This is important new information, as it contrasts with the cytolytic activities of both cell populations (recall Fig 5B and 5C), indicating that *in vitro* cytolytic activity against IFN-γ pre-treated, infected cells is not reliably predictive for an antiviral function *in vivo*.

### TCR-transduced CD4 T cells enhance the antiviral efficacy of TCR-transduced CD8 T cells

Interestingly, CD4-TCR_NLV_ cells, though not antiviral effectors on their own (see above), enhanced the antiviral function of CD8-TCR_NLV_ cells when co-administered as a 1:4 mixture of 2 x 10^6^ non-protective CD4-TCR_NLV_ and 8 x 10^6^ protective CD8-TCR_NLV_ cells (spleen, P < 0.001; lungs, P = 0.009; liver, P = 0.049). Specifically, at least in spleen and lungs, this mixture turned out to be more efficient than the higher number of 10^7^ protective CD8 T cells (P = 0.037 and P = 0.005, respectively), which indicates synergism in the sense of a helper effect of the CD4 T cells. Protection by the mixture was clearly NLV epitope-specific, as any antiviral function was abolished when the NSG/HHD mice were infected with the antigenicity-loss mutant mCMV-NLV_Ala_.

Antiviral control, determined on day 11, should be preceded by migration of transferred cells into the tissues. As shown for spleen and liver (S5 Fig), CD8 and CD4 T cells could be recovered on day 3 after intravenous cell transfer from spleen leukocyte and liver non-parenchymal cell populations. Notably, after transfer of 10^7^ CD8-TCR_NLV_ cells in absence of CD4-TCR_NLV_ cells, recovery of CD8-TCR_NLV_ cells from the tissues was poor but was enhanced after transfer of the mixture. Thus, apparently, the CD4 T cells’ helper effect is by facilitating tissue infiltration, as limited longevity of transferred CD8-TCR_NLV_ cells should not yet be an issue in the first 3 days.

### NLV-specific human T cells prolong the survival of mCMV-NLV-infected HLA-A2.1 transgenic NSG/HHD mice by delaying viral pathology

By design, the model’s strength is to test immediate effector functions of the transferred T cells, as their longevity in the NSG/HHD recipient is limited by the constitutive absence of IL-2/4/7/9/15/21 signaling and cytokine heterology, except for single doses of IL-2 and IL-7 coadministered with the human T cells. Furthermore, the model of a genetically, and thus enduringly, immunodeficient recipient is highly demanding for completeness of virus eradication at an early stage, as it is established experience that, in the long run, a few infectious units (just 1–5 PFU) of ‘therapy escapee virus’ will eventually cause death in NSG/HHD and related, immunodeficient mouse strains based on tissue pathology from exponential cytopathogenic viral spread over time [71].

**Fig 7:**
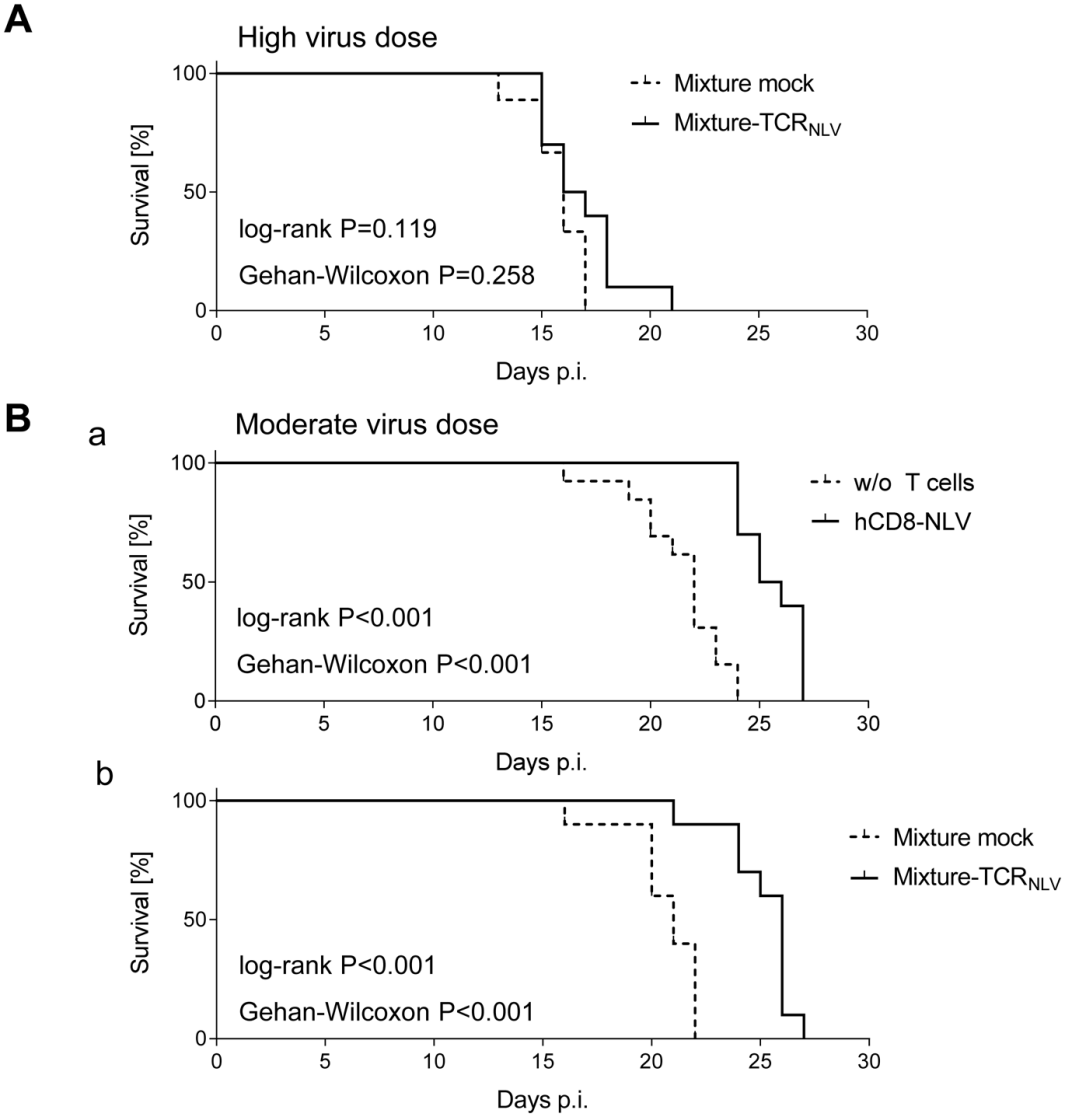
Survival of mCMV-NLV-infected NSG/HHD mice upon adoptive transfer of NLV-specific human T cells. Groups of 10 γ-irradiated (2 Gy) NSG/HHD mice were infected (A) with 1x10^5^ PFU or (B) with 1x10^3^ PFU of mCMV-NLV, and in (A; B,b) they received the 1:4 mixtures (see the legend to Fig 6) of TCR_NLV_-transduced (solid graphs) or mock-transduced (dashed graphs) human CD4 and CD8 T cells on the day of infection as a pre-emptive therapy. (B,a) As a reference for comparison, recipients received 1x10^7^ cells of CTLL hCD8-NLV (solid graph) or were left with no T cell transfer (dashed graph, w/o T cells). Survival rates over time are displayed as Kaplan Meier survival plots. Statistical significance of differences in survival was calculated using the log-rank test and the Gehan-Wilcoxon test. In the most efficient therapy with the mixture of TCR_NLV_-transduced CD4 and CD8 T cells (B,b), the median survival time was 26d (range: 21-27d) compared to 21d (range 16-22d) in the mock-transfected control group.

Despite these inherent constraints, transfer of human NLV-specific T cells led to prolonged survival, which was modest at very high initial virus burden and did not reach statistical significance (Fig 7A) but was highly significant (P < 0.001 by log-rank test as well as by Gehan-Wilcoxon test) at a moderate, more realistic, initial virus dose (Fig 7B; see Discussion). Specifically, hCD8-NLV cells delayed mortality when compared to the no transfer group (Fig 7B and 7a), and the 1:4 mixture of TCR_NLV_-transduced CD4 and CD8 T cells delayed mortality when compared to the mock-transduced mixture lacking an NLV-specific TCR (Fig 7B and 7b).

**Fig 8:**
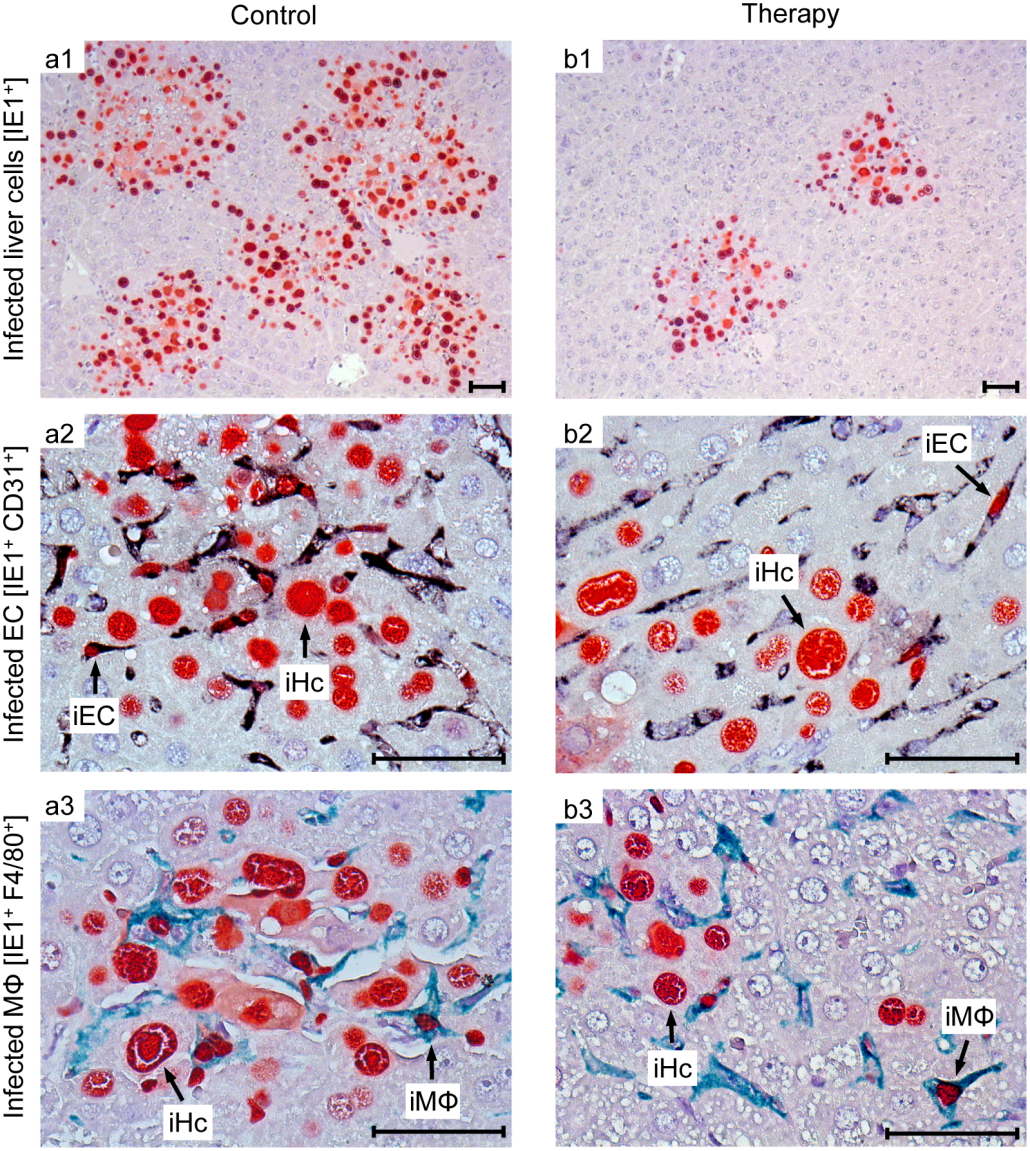
Reduced viral spread and histopathology in liver tissue, differentiated by infected cell type, as correlate of protection by human TCR_NLV_-transduced T cells. Corresponding to the survival data (Fig 7B and 7b), liver tissue sections were taken on day 20 after infection with 10^3^ PFU of mCMV-NLV and adoptive transfer of mock-transduced (group: Control) or TCR_NLV_-transduced (group: Therapy) human T cells, each composed of 2x10^6^ CD4 and 8x10^6^ CD8 cells. Low-magnification IHC images show overviews of the extent of tissue infection (a1,b1; plaque-like foci of infected, red-stained IE1^+^ liver cells), and higher-magnification 2D-IHC images reveal infection of hepatocytes (iHc), which are distinctive by cytomorphology (red staining of intranuclear IE1 protein), combined with infected endothelial cells (a2, b2; iEC, co-expressing red-stained IE1 protein and black-stained CD31 antigen) or infected macrophages (a3,b3: iMΦ, co-expressing red-stained IE1 protein and turquoise green-stained F4/80 antigen). Bar markers represent 50 μm throughout.

**Fig 9:**
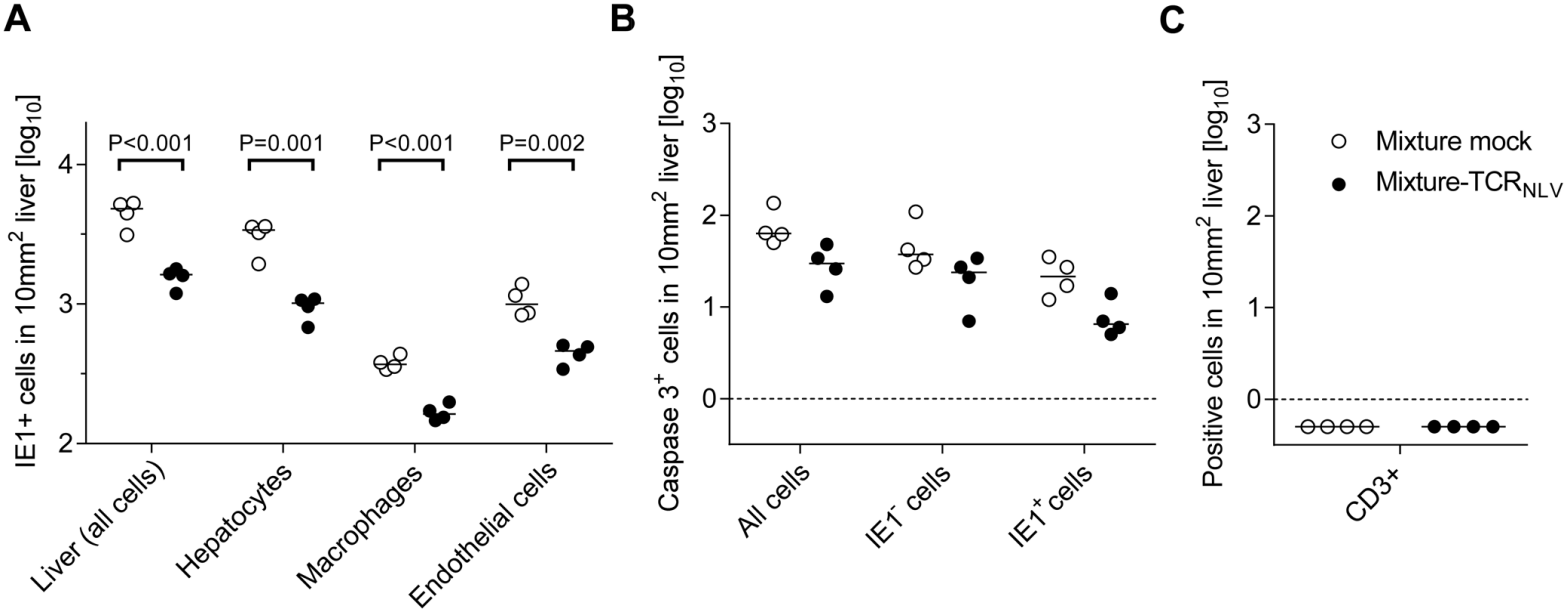
Immunohistological quantitation of infected and of apoptotic liver cells. (A) Corresponding to the representative IHC images shown in Fig 8, infected cells were counted in absolute numbers on day 20, differentiated by cell type as indicated, in representative 10-mm^2^ areas of liver tissue sections. Symbols represent cell counts from individual mice, with the median values marked. Open circles, mice from the mock-transduction control group; closed circles, mice from the TCR_NLV_-transduction therapy group. (B) Corresponding to the representative IHC images shown in Fig 10 and S6 Fig, apoptotic (caspase 3^+^) liver cells were counted on day 20 in absolute numbers, differentiated by infected (IE1^+^) and uninfected (IE1^-^) liver cells. Further details as in subfigure (A). The calculation of P values for the significance of the observed trend to a reduction in apoptosis events in the therapy group makes no sense in view of the scientific question of the experiment, namely if xenogeneic T cells exert an immunopathology indicated by enhanced apoptosis, which was obviously not the case. Subfigure (C) points to the fact that on day 20 after infection and cell transfer, T cells (black staining of CD3ε antigen as in Fig 4) were no longer present in either experimental group. Symbols as in subfigure (A).

To demonstrate a histopathological correlate of mortality, we performed immunohistochemical (IHC) imaging for livers from the mock-transduction control group and the therapy group (corresponding to Fig 7B and 7b) at the time of onset of mortality (day 20) in the control group (Fig 8). Overview sections, identifying infected liver cells by their expression of intranuclear viral IE1 protein (Fig 8a1 and 8b1; red staining), reveal extended plaque-like lesions from the cytopathogenic effect of virus replication in the control livers, whereas foci of infection were less frequent and less extended in the therapy group, though, as predictable from exponential (log-linear) growth ([36,37] and references therein) tissue infection is likely to close up to the control group with a few days delay explaining delayed onset of mortality in the therapy group. Higher-magnification 2-color IHC images show infected hepatocytes (iHc, red staining of IE1), which are distinctive by cytomorphology, infected endothelial cells (iEC, red staining of IE1 and black staining of CD31 antigen) (Fig 8a2 and 8b2), and infected macrophages (iMΦ, red staining of IE1 and turquoise-green staining of F4/80 antigen) (Fig 8a3 and 8b3). Quantification by cell counting reveals significant reductions in the numbers of infected cells in the therapy group, with no notable cell-type specific preferences (Fig 9A).

**Fig 10:**
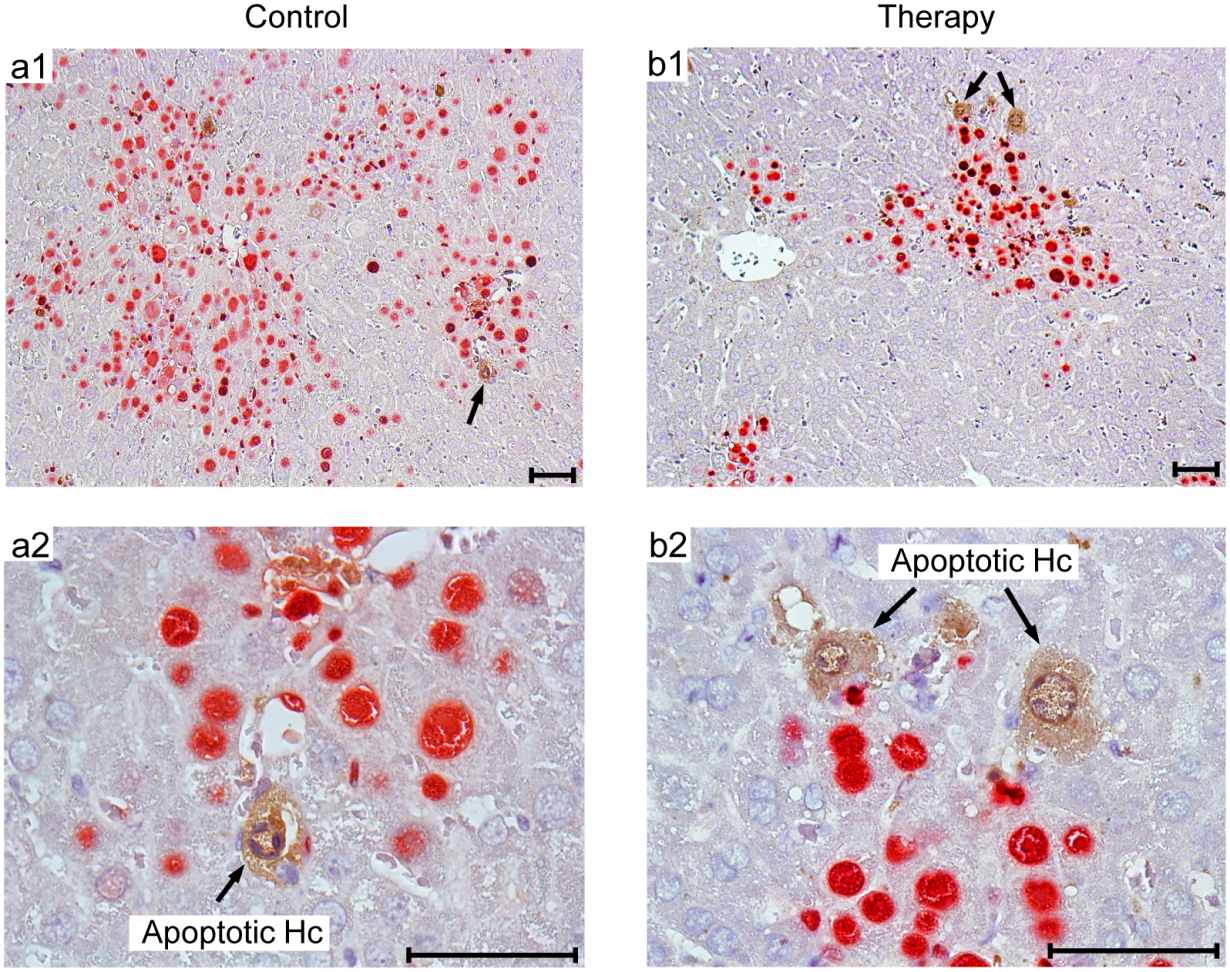
Human NLV-specific T cells do not exert an immunopathology in terms of enhanced apoptosis or necrotic/necroptotic lesions outside of foci of infection. Corresponding to the quantitative data provided in Fig 9B, representative 2C-IHC images of liver tissue sections show uninfected, apoptotic hepatocytes (IE1^-^caspase 3^+^, brown cytoplasmic staining), exclusively within IE1^+^ (red stained) foci of infection. (a1, b1) Low-magnification images for an overview of viral histopathology. Arrows in these overview images point to regions resolved to greater detail (a2, b2). Note that infected, apoptotic cells are difficult to find coincident with uninfected, apoptotic cells, because of the generally low incidence of apoptosis in this cell transfer model. An example for the existence of infected, apoptotic cells is shown in S6 Fig. Bar markers represent 50 μm throughout.

Although therapeutic cells equipped with a transgenic virus epitope-specific TCR are not expected to cause an immunopathology, there was residual concern that xenogeneic graft-versus-host interactions, possibly by the endogenous TCRs or by TCR-independent mechanisms, might cause immunopathology and thus obscure the results of the model, in particular the survival data. We have therefore compared general histopathology and caspase 3-dependent apoptosis in liver tissue sections from the control group and the therapy group at the time of onset of death in the control group (day 20 in this specific experiment) by 2-color IHC staining of infected cells (IE1^+^, red staining) and apoptotic cells (caspase-3^+^, brown staining) (Fig 10). Notably, histopathology proved to be confined to the foci of infection, as no lesions or necrotic areas were observed in tissue regions not yet reached by the infection. Interestingly, apoptotic cells were predominantly uninfected Hc (IE1^-^caspase-3^+^), though localizing to the foci of infection. This suggests an apoptosis induction *in trans*, but apoptotic iHc (IE1^+^caspase-3^+^) can also be found occasionally (IHC image in S6 Fig and quantitation in Fig 9B). The relatively low number of apoptotic infected cells relates to the known fact of cell death-inhibiting viral genes being expressed in CMV-infected cells (for reviews, see [72,73]. Most relevantly, however, the number of apoptotic cells, even that of uninfected apoptotic cells, was definitively not increased but rather tended toward being reduced in the therapy group (Fig 9B), a finding that rules out any relevant immunopathology by the xenogeneic cell transfer in this immunotherapy model. As reported recently, mCMV infection as such does not induce immunopathology mediated by CD8 T cells, except if the host is combined-deficient in NK cells and perforin [74]. Finally, IHC specific for CD8 did no longer reveal presence of transferred CD8 T cells in the liver on day 16 (Fig 9C), reflecting their limited longevity due to the multiple interleukin-signaling deficiency in NSG/HHD mice. In conclusion, histopathology is undoubtedly of viral etiology and is clearly reduced by antiviral human CD8 T-cell immunotherapy.

## Discussion

We herein describe a new mouse model mimicking pre-emptive adoptive T-cell therapy of systemic HCMV infection in immunocompromised patients. The model is based on HLA-A2.1 transgenic NSG/HHD mice that are first infected with chimeric virus mCMV-NLV and are subsequently infused with human T cells specific for the immunodominant HLA-A2.1-restricted NLV epitope of HCMV pp65 (UL83). Despite the species barrier, transferred human T cells formed protective NIF, controlled virus spread, and limited viral pathology in classical organs of CMV disease manifestations without mediating immunopathology, thereby conveying a significant survival benefit to infected mice.

Previous mouse models for HCMV infection utilized transplantation of HCMV-infected human cells, e.g. fibroblasts embedded in agarose plugs [75], glioblastoma cell lines [76], and hepatocytes [23] or, alternatively, human fetal thymus/liver and retinal tissues infected with HCMV [19–21]. Although having contributed important information, implant models, however, are not designed for studying virus dissemination to functional organs and virus spread in these organs that causes histopathology resulting in morbidity and mortality. Therefore, life-saving therapeutic interventions could not be evaluated in such models. In contrast, systemic infection of NSG/HHD mice with the herein described chimeric virus mCMV-NLV provides a model that—in a reasonably good first approximation—resembles relevant aspects of HCMV disease in HSCT recipients, including the infection of vital organs involved in virus-associated morbidity and cause of death. It should be emphasized that the NLV peptide was genetically engineered into the non-essential IE2/m128 protein of mCMV. Thus, its synthesis and presentation occurs according to the regular gene expression kinetics of the viral IE2 carrier protein.

The NLV peptide was here just chosen as a paradigm. The model is meant as a modular system that can easily be adapted to scientific and medical needs. Specifically, by analogous strategy, the NLV peptide can be replaced with any other HLA-presented HCMV epitope of interest or might be combined with additional epitopes integrated at other positions of the mCMV genome [31]. Such chimeric viruses, together with various HLA transgenic NSG mouse strains, promise to allow analysis of the broad repertoire of HLA restricted T-cell responses against HCMV ([77], reviewed in [78,79]).

Importantly, like for HCMV infection of human cells, HLA-A2.1/NLV-antigen presentation was shown here to be susceptible to immune evasion mediated by virally encoded proteins that inhibit the trafficking of peptide-loaded MHC-I molecules to the cell surface [49,80–84]. Although the proteins involved and the precise molecular mechanisms by which they mediate immune evasion differ between individual CMV species, the common biological outcome is inhibition of antigen presentation, which has been shown to reduce but not to preclude a protective effect of adoptively transferred murine CD8 T cells in immunocompromised mice ([85,86], reviewed in [7–9,49,84]). Likewise, contrasting with earlier antigen presentation studies in transfected cells, HCMV ‘immune evasion’ genes gpUS2-11 also fail to completely prevent antigen presentation in infected cells [38,66]. In this context it is important to note that in the here presented model the species origin of the transferred CD8 T cells, secreting murine or human IFN-γ, appeared not to be qualitatively critical for controlling mCMV-NLV infection despite immune evasion mechanisms of mCMV being active that are relieved by murine IFN-γ [47,48]. For the future, the modular model is open for the option to further ‘humanize’ the conditions by replacing immune evasion genes of mCMV with those of HCMV in a next generation of chimeric mCMV-NLV viruses.

A concern affecting the validity of the model has been a potential species barrier for an efficient infiltration of infected murine tissues by human CD8 T cells. It is therefore important to recall that we observed tissue infiltration and epitope-specific formation of protective NIF by NLV-specific human CD8 T cells, reduction of viral load in representative murine target organs of CMV disease, and prolonged survival. The data in the model thus agree with previous work in humans showing that adoptive transfer of NLV-specific CD8 T cells is beneficial in patients undergoing allogeneic HSCT [87–89]. Importantly, a concern regarding a putative immunopathology by transferred xenogenic TCR-transduced T cells could be invalidated in the liver by absence of tissue necrosis and of apoptotic cells in tissue areas outside of the foci of infection.

Although previous studies have highlighted the importance of CD4 T-helper cell support in HSCT patients [87,90–93], antiviral T-cell immunotherapies so far mainly focused on cytolytic CD8 T cells. A helper contribution by CD4 T cells was proposed to be needed primarily for longevity of transferred CD8 T cells mediating enduring protection. Applying the here described new model for testing the requirements of immunotherapy by TCR_NLV_-transduced human CD8 T cells as a model for an HCMV seronegative HSCT donor, CD4 T cells indeed supported CD8 T cell-mediated antiviral control in mCMV-NLV-infected NSG/HHD mice, while not exerting antiviral effector functions on their own. As an interesting new information, this ‘helper’ effect corresponded to an enhanced early tissue infiltration by the CD8 T cells, indicating that CD4 T cell help is beneficial already at an early stage of immunotherapy and not only at late stages.

In the current version of the new model, the adoptive transfer of a single dose of human T cells resulted in epitope-specific tissue infiltration and tissue persistence of T cells within NIF structures for at least 11 days, and in a reduction of virus spread associated with confinement of the infection within NIF. At a very high dose of intraplantar infection, however, the survival benefit was insignificant, whereas at a moderate initial virus dose the survival benefit, though now statistically highly significant, did not result in cured long-term survivors, which corresponded to absence of tissue-infiltrating progeny of transferred T cells and relapse of virus spread at the time of onset of mortality.

For the interpretation of the relevance of only delayed but not prevented mortality, several aspects in which the model by design differs from the clinical correlate of HSCT-associated HCMV reactivation need to be considered: (i) in the model the virus doses used are much higher than to be expected in a clinical setting where acute primary infection of a seronegative HSCT recipient (R^-^) is a rare event, while reactivation in a seropositive, latently infected recipient (R^+^) likely originates from very few cells, if not just from one single cell. mCMV latency models indicated rare and stochastic reactivation events under immunocompromised conditions *in vivo* [94,95] as well as after tissue explantation ([96,97], reviewed in [41,42]). Indeed, it is clinical routine to start antiviral therapy, not just immunotherapy but also therapy with antiviral drugs, upon first detection of viral DNA by highly-sensitive PCR monitoring when the load of infectious virions is still minimal and mostly undetectable. This strategy is known as pre-emptive therapy, and has recently been reviewed in a clinical article by Seo and Boeckh [98]. Along the same line of argument, in approved HCMV vaccine trials, minimal doses of challenge virus (as low as 10 infectious units) were actually used to clinically evaluate vaccination efficacy (reviewed in [99]), a fact that not many are aware of. (ii) the model, by design, measures immediate antiviral effector functions of the transferred cells, as the NSG/HHD host does not support longevity and expansion of transferred cells due to multiple genetic deficiency in common γ-chain-dependent interleukin signaling, and (iii) in the clinical situation of pre-emptive immunotherapy, and likewise also of pre-emptive antiviral drug therapy, of HSCT-associated HCMV reactivation, the medical demand is only to bridge the critical phase of transient immunodeficiency until reconstitution of intrinsic host immunity in consequence of HSCT takes over. Such a temporary, transferred protection likely requires much lower cell numbers [63]. In contrast, in the model, as it currently stands, the NSG/HHD host is constitutively, and thus enduringly, immunodeficient so that even a single infectious unit of a ‘therapy escapee virus’ eventually causes death by viral tissue pathology. With these arguments in mind, the model provides a rigorous ‘stress test’ for the quality of adoptively transferred antiviral T cells, and the prolongation of host survival observed in this rigorous model predicts a good functionality of the tested human T cells in the less demanding clinical reality of low-dose HCMV reactivation followed by reconstitution of intrinsic antiviral immunity.

We wish to emphasize that the here introduced model should be understood as a basic module of a modular model system that is open to be developed further by ourselves and, hopefully, joined by other investigators. An obvious next step could be to improve the longevity and promote clonal expansion of adoptively transferred T cells by prolonged substitution with IL-2 and IL-7, and to include also IL-15 [100]. Another option for adaptation of the model to clinical needs could be the transfer of defined T-cell subsets with stem cell properties [101]. Finally, and most obviously, NSG/HHD adoptive transfer recipients could be further ‘humanized’ by human CD34^+^ stem-cell transplantation to reconstitute them with human immune system for the priming of intrinsic antiviral effector and helper cells [102,103] that take over for enduring viral control after the transient protection by adoptive immunotherapy has prevented life-threatening early-onset CMV disease.

In conclusion, we introduce here a promising mouse model of systemic CMV infection that allows the direct analysis of antiviral activity of HCMV-specific T-cell products in terms of infection control in functional organs and survival benefit. The model has best prospects to stimulate future research aimed at optimizing it for clinical needs, and has great potential to expedite the development of improved adoptive cell transfer as well as vaccine strategies against HCMV infection.

## Material and Methods

### Mice, cells, and viruses

C57BL/6 and NOD.Cg-Prkdc^scid^ Il2rg^tm1Wjl^ Tg(HLA-A/H2-D/B2M)1Dvs/SzJ (NSG/HHD) [27] mice were bred and maintained under SPF conditions in the Central Laboratory Animal Facilities at the University Medical Center Mainz and at the University Hospital of Regensburg. NSG/HHD were purchased from Jackson Laboratory (Bar Harbor, ME, USA). Mice were sacrificed by CO_2_ inhalation or cervical dislocation. Primary mouse embryonic fibroblasts (MEF) from C57BL/6 and NSG/HHD mice were generated by standard methods [104] and maintained in minimal essential medium (MEM) supplemented with 10% fetal calf serum. HLA-A2.1^+^ primary human foreskin fibroblasts (HFF) were grown in MEM supplemented with 10% fetal calf serum, 2 mM l-glutamine, 50 mg/L gentamicin and 0.5 ng/ml basic fibroblast growth factor (Life Technologies, Darmstadt, Germany) [66]. Human CD4 and CD8 T cells were isolated from buffy coat products of either HLA-A2.1^+^ CMV-seronegative or—seropositive healthy donors using immunomagnetic bead technology (Miltenyi Biotec, Bergisch Gladbach, Germany). Bacterial artificial chromosome (BAC)-derived virus mCMV-Δm157 [105] as used as non-chimeric reference virus. Virus stocks of mCMV-Δm157, mCMV-WT.BAC [106], and mCMV-Δm04/m06/m152 (mCMV-ΔvRAP) [50,51] were prepared from infected C57BL/6 MEF by sucrose-gradient ultracentrifugation as described [107]. HCMV immune evasion gene (US2,3,6,11) deletion mutant RV-KB6 and the combined US2,3,6,11, and pp65/UL83 deletion mutant RV-KB15 were described previously ([66] and [16], respectively).

### Ethics statement

Animal research protocols of the University Medical Center Mainz were approved by the ethics committee of the Landesuntersuchungsamt Rheinland-Pfalz, permission numbers 23177-07/G09-1-004 and 23177-07/G11-1-004, according to German Federal Law §8 Abs. 1 TierSchG (animal protection law). Human blood cells were isolated from buffy coat products of healthy donors after written informed consent and approval by the ethics committee of the Landesärztekammer Rheinland-Pfalz and University Hospital of Regensburg (permission number 837.149.10 and 13-101-0240, respectively) and performed according to the Declaration of Helsinki.

### Insertion of the HCMV epitope pp65_495-503_ NLVPMVATV into the mCMV genome

For generating mCMV-NLV, 69 nucleotides of HCMV ORF *UL83* (n119,567-n119,499; GenBank accession no. X17403) were integrated at nucleotide position n186,511 into ORF*m128* of the mCMV genome (GenBank accession no. NC_004065). These nucleotides code for 23 amino acids including the NLV epitope and its natural flanking regions. The mutagenesis was performed essentially as previously described [50]. A linear PCR fragment containing a kanamycin resistance gene flanked by two FRT sites and viral homologies of ORF*m128* was generated by PCR with primers m128_NLV_for (5′-acg tcg ggc aga aag ctg ggt tat ctc gac gtg gcg gag aag atc ctg gcc cgc aac ctg gtg ccc atg gtg gct acg gtt cag ggt cag aat ctg aag tac cag gaa ttc agg acg acg acg aca agt aa-3`) and m128_NLV_rev (5′-gga tca cgc cga gaa cct cga ggg gac cgt tgc aca tgg ggt att cct tgc gca gca gga aca ctt aac ggc tga-3′) and plasmid pKD46 [108]. This fragment was inserted into the BAC plasmid pΔm157 [105] by homologous recombination in *E*. *coli*. After subsequent FLP-mediated excision of the resistance gene, the correct nucleotide insertion was verified by sequencing (GATC, Freiburg, Germany). The generation of mCMV-NLV_Ala_ was performed as described above except using primer m128_NLV_Ala__for (5′-acg tcg ggc aga aag ctg ggt tat ctc gac gtg gcg gag aag atc ctg gcc cgc aac ctg gtg ccc atg gtg gct acg gca cag ggt cag aat ctg aag tac cag gaa ttc agg acg acg acg aca agt aa-3`) instead of m128_NLV_for. Recombinant CMVs were reconstituted by transfection of purified BAC DNA into C57BL/6 MEF, and high titer virus stocks were purified as described [107].

### Flow cytometry, antibodies, and peptides

Flow cytometry was performed on FACS Canto II or FACS LSR II (BD Biosciences, Heidelberg, Germany). Fluorochrome-labeled monoclonal Antibodies (mAb) were anti-mouse H-2K^b^ (clone AF6-88.5), CD62L-PE-Cy7 (clone MEL-14), CD8-FITC (clone 53–6.7), anti-human CD8-PerCP (clone SK1), CD8-APC (clone RPA-T8), CD4-APC (clone RPA-T4), CD28-FITC (clone CD28.2), CD95-PE (clone DX2), CD45RA-PE (clone HI100), CD45RO-PE (clone UCHL1), CD62L-FITC (clone DREG-56), HLA-A2.1-PE (clone BB7.2) (all BD Biosciences), anti-mouse CD44-PB (clone IM7) (BioLegend, San Diego, CA, USA), and anti-human CCR7-FITC (clone 150503) (R&D Systems, Minneapolis, MN, USA). Intracellular staining of MEF cells was performed with m164/gp36.5 antiserum [51], followed by Alexa Fluor 488-conjugated mAb goat anti-rabbit IgG (Life Technologies, Darmstadt, Germany). Fluorochrome-labeled HLA-A2.1/NLV tetramer was synthesized by Beckman Coulter (Krefeld, Germany). Peptides were synthesized by PSL (Heidelberg, Germany). Analyses were performed with software FlowJo 7.6.5 (Tree Star, Ashland, OR, USA).

### T-cell stimulation and assays

Human NLV-specific CD8 T-cell lines were expanded from purified CD8 T cells of HLA-A2.1^+^ HCMV-seropositive healthy donors by stimulation with pp65 (UL83)_495-503_ NLV peptide (10^−6^ M)-loaded and irradiated (35 Gy) autologous peripheral blood mononuclear cells (PBMC) over 2 weeks at the T cell-to-PBMC ratio of 1:1 in AIM-V medium (Life Technologies). AIM-V was supplemented with 10% human serum, recombinant human interleukin (rhIL)-2 (50 IU/mL; Proleukin, San Diego, CA, USA), rhIL-7, and rhIL-15 (each 5 ng/mL; R&D Systems) (AIM-V^cytokine^). A murine NLV-specific CD8 T-cell line was generated and weekly restimulated as previously described [38]. MEF were infected with mCMV under conditions of centrifugal enhancement of infectivity [104]. HFF were infected with HCMV-RVKB6 and—RVKB15 for 24h at an MOI of 5. Standard 4h [^51^Cr]-release and 20h interferon (IFN)-γ ELISpot-assays were performed in duplicates as reported [109,110]. Dose-escalating equilibrium tetramer binding data were plotted in Scatchard analysis of mean fluorescence intensity (MFI)/concentration of tetramer against MFI. The dissociation constant K_D_ equals -1/slope [44,45].

### TCR genes and retroviral transduction

TCR_NLV_ is a codon-optimized and affinity fine-tuned variant of the previously described TCR AV18/BV13 [16,111]. A short self-cleaving F2A sequence [112] was used to link the N-terminus of TCRα chain to the C-terminus of TCRβ chain by ligation PCR technology [113]. The antisense oligonucleotide sequence was 5’-atg gct atg gtg aag cgg aag gac ttc gtg aaa caa acg ttg aat ttt gac ctt ctc aag ttg gcc gga gac gtg gag tcc aac ccc ggg cct atg gag aag aac ccc ctg gcc gcc ccc-3’. The coding sequence of the TCRβ-F2A-TCRα gene construct was inserted into the multiple-cloning-site of the drug-selectable retroviral vector pMX (BioCat, Heidelberg, Germany). Purified CD4 and CD8 T cells from HCMV-seronegative donors were pre-stimulated with anti-CD3/CD28 Dynabeads (Life Technologies) in AIM-V medium (supplemented with 100 IU/mL rhIL-2) and then retrovirally transduced with TCR_NLV_ as previously described [113]. Following drug-selection on TCR_NLV_^+^ cells, T cells were expanded *in vitro* for a period of 7-12d, using anti-CD3/CD28 beads (3–5 μL/10^6^ cells) in AIM-V^cytokine^, prior to their adoptive transfer.

### Quantitation of tissue infiltration and *in vivo* antiviral function of HCMV epitope-specific T cells

Eight- to 10-week-old NSG/HHD mice were sublethally (2 Gy) γ-irradiated for cell homing conditioning, followed by intraplantar infection with 1x10^5^ PFU of the indicated viruses. Subsequently, a single dose of up to 1x10^7^ human or murine T cells was injected intravenously. Human T cells were co-injected with rhIL-2 (1000 IU/mouse) and FcIL-7 (20 μg/mouse; Merck, Darmstadt, Germany). At indicated times post-infection, virus replication in spleen, lungs, and liver was assessed by quantitation of infectivity from the respective organ homogenates in a virus plaque assay (PFU assay) [107]. Infected cells and T cells in liver tissue sections were visualized simultaneously in their microanatomical context, specifically in nodular inflammatory foci (NIF), and quantitated by two-color immunohistochemistry (2C-IHC) specific for the intranuclear viral IE1 protein and a conserved CD3ε epitope [107]. For testing maintenance of transferred human T cells, flow cytometric analysis was performed on splenocytes retrieved by standard methods and on non-parenchymal liver cells isolated as described [97].

### 2-color immunohistochemical (2C-IHC) analyses of infection, differentiated by cell type, and of apoptosis

To detect any type of mCMV-infected cells, one-color IHC specific for the intranuclear viral protein IE1 was performed on liver tissue sections as described in detail elsewhere [107]. For differentiating infected cells by cell type, 2C-IHCs were performed by combining IE1-specific labeling with the labeling of cell type-specific markers, such as CD31 for the identification of endothelial cells (EC) or F4/80 (Ly71) antigen for the identification of macrophages (MΦ), essentially as described recently for a 3C-IHC analysis that has included the same markers [36]. 2C-IHC specific for IE1 and active caspase 3 was described previously [114] and applied with modifications. In brief, intranuclear IE1 protein was labeled with monoclonal Ab CROMA 101 and stained in red with alkaline phosphatase-1-conjugated polyclonal goat anti-mouse IgG (AbD Serotec, Puchheim, Germany) and fuchsin substrate-chromogen kit 2 (Dako-Cytomation, Hamburg, Germany). Active caspase 3 was detected with rabbit anti-active caspase 3 IgG (Biovision, Milpitas, CA, USA) and stained in brown by using the ImmPRESS reagent: anti-rabbit-Ig-peroxidase (Vector Laboratories, Peterborough, UK) with 3,3’-diaminobenzidine (DAB) as substrate. A light blue counterstaining was achieved with hematoxylin.

### Statistical analyses and calculation of viral doubling times in host tissues

Statistical significance of differences between two independent data sets was evaluated by two-sided unpaired t test with Welch’s correction of unequal variances. Comparison of survival curves was performed with log-rank test and Gehan-Wilcoxon test. Differences were considered statistically significant for P values of <0.05. Viral doubling times (vDT = log2/*a*) and the corresponding 95% confidence intervals were calculated by linear regression analysis from the slopes *a* of log-linear growth curves [36]. All analyses were performed with Graphpad Prism 6.04 (GraphPad Software, San Diego, CA, USA).

## Supporting Information

S1 Fig Phenotypic characterization of NLV-peptide specific murine and human CD8 T cells. (A) Polyclonal murine CD8 T cells gated from C57BL/6 splenocytes (left panel) and NLV-peptide specific hCD8/mCD8^+^ cells of T cell line mCD8-NLV (right panel) were analyzed for expression of CD44 and CD62L to identify CD44^lo^CD62L^hi^ naive (T_N_) and CD44^hi^CD62L^hi^ central memory (T_CM_) T cells as well as CD44^hi^CD62^lo^ effector (T_E_) and effector-memory (T_EM_) T cells. (B) Cells of the NLV-peptide specific human CD8 T-cell line hCD8-NLV were stained for the expression of CD28 and CD95/Fas to identify CD28^+^CD95^low^ T_N_, CD28^+^CD95^high^ T_CM_, and CD28^-^CD95^high^ T_EM_ subsets. Further characterization of the phenotype of hCD8-NLV cells included the T-cell differentiation markers CD45RA, CD45RO, CD62L, and CCR7.
(TIF)

S2 Fig Immune evasion molecules of mCMV target human HLA-A2.1 for downmodulating its cell surface expression. MEF derived from NSG/HHD mice were pre-treated with IFN-γ for 48h and infected with the indicated viruses. Shown are cytofluorometric 2D dot plots of cell surface MHC-I expression (H2-K^d^, upper panel; HLA-A2.1, lower panel; abscissa: PE-fluorescence intensity) depending on the infection of cells indicated by expression of the intracellular infection marker gp36.5/m164 (ordinate; Alexa Fluor488-fluorescence intensity). Arrows point to the infected gp34/m164^+^ cell population that is MHC-I^low^ after infection with mCMV-WT.BAC and MHC-I^high^ after infection with mCMV-ΔvRAP, in which genes encoding the viral regulators of antigen presentation (vRAP) gp34/m04, gp48/m06, and gp40/m152 are deleted. Data are representative of two independent experiments.
(TIF)

S3 Fig Cytolysis of HCMV-infected human fibroblasts by TCR_NLV_-transduced CTL. Immunomagnetically-selected human CD8 T cells were retrovirally transduced with TCR_NLV_ (CD8-TCR_NLV_, filled circles) or empty vector (CD8 mock, open circles). After *in vitro* expansion with anti-CD3/CD28 beads for a period of 10d, cells were analyzed at the indicated effector-to-target (E:T) cell ratios for cytolysis of HLA-A2.1^+^ human primary foreskin fibroblasts infected with HCMV immune evasion gene deletion mutant RVKB6 (NLV^+^) or with the combined immune evasion and pp65/UL83 deletion mutant RVKB15 (NLV^-^). Data represent means of duplicate assay cultures. Error bars indicate the range.
(TIF)

S4 Fig Phenotypic characterization of TCR_NLV_-transduced CD8 and CD4 T-cell subsets. Immunomagnetically selected CD8 (A) and CD4 (B) T cells were stained for cytofluorometric phenotyping before (upper panels) and after retroviral transduction with TCR_NLV_ (CD8-TCR_NLV_ and CD4-TCR_NLV_ cells, respectively) followed by *in vitro* expansion for a period of 10d (lower panels). Cell surface markers CD28 and CD95/Fas identify CD28^+^CD95^low^ naive (T_N_), CD28^+^CD95^high^ central memory (T_CM_), and CD28^+^CD95^high^ effector-memory (T_EM_) T cells. Further characterization of the phenotype included the T-cell differentiation markers CD45RA, CD45RO, CD62L, and CCR7. Shown are 2D dot plots. Percentages and mean-fluorescence intensities (MFI) of labeled cells are indicated.
(TIF)

S5 Fig CD4-TCR_NLV_ cells function as helper cells by enhancing early organ recruitment of CD8-TCR_NLV_ cells. Corresponding to data on the control of organ infection (see the legend of Fig 6), adoptively transferred CD8-TCR_NLV_ cells (left panels) and CD4-TCR_NLV_ cells (right panels) were analytically retrieved by cytofluorometric analysis from spleen (upper panels) and liver (lower panels) of NSG/HHD mice on day 3 after intraplantar infection with 1x10^5^ PFU of mCMV-NLV. Grey-shaded bars: retrieval after transfer of 1x10^7^ CD8-TCR_NLV_ cells. Black bars: retrieval after transfer of a mixture consisting of 2x10^6^ CD4-TCR_NLV_ and 8x10^6^ CD8-TCR_NLV_ cells. Bars represent mean % values of data from three individual mice. Error bars indicate SEM. P values for significance of differences were calculated by using the ratio paired t-test.
(TIF)

S6 Fig Detection of infected, apoptotic hepatocytes in liver tissue sections. Corresponding to the 2C-IHC analysis of liver tissue infection and apoptosis shown in Fig 10, where uninfected, apoptotic hepatocytes (Hc) were found to be located in foci of infection, images here show an example of an infected, apoptotic hepatocyte (iHc) identified by co-expression of intranuclear IE protein (red staining) and cytoplasmic active caspase 3 (brown staining). (a1) overview of liver tissue infection; the arrow points to a region that is resolved to greater detail in the higher magnification image (a2). Bar markers: 50 μm.
(TIF)
