# Supplementary figures and images for: Evaluating Human T-Cell Therapy of Cytomegalovirus Organ Disease in HLA-Transgenic Mice
Source: PLoS Pathog. 2015 Jul 16;11(7):e1005049. doi: 10.1371/journal.ppat.1005049 (PMC4504510; doi:10.1371/journal.ppat.1005049)

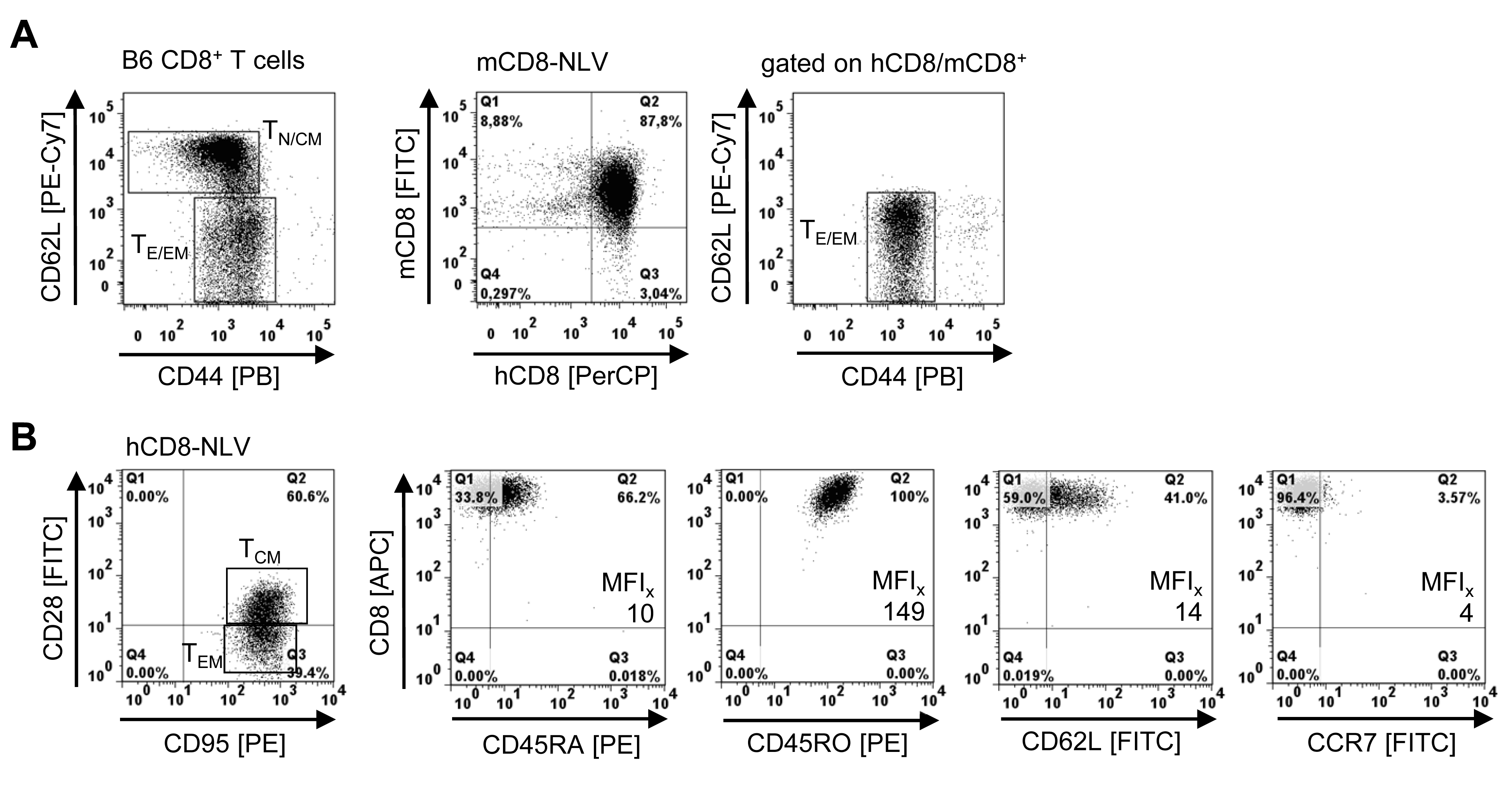

Supplement: S1 Fig — (A) Polyclonal murine CD8 T cells gated from C57BL/6 splenocytes (left panel) and NLV-peptide specific hCD8/mCD8+ cells of T cell line mCD8-NLV (right panel) were analyzed for expression of CD44 and CD62L to identify CD44loCD62Lhi naive (TN) and CD44hiCD62Lhi central memory (TCM) T cells as well as CD44hiCD62lo effector (TE) and effector-memory (TEM) T cells. (B) Cells of the NLV-peptide specific human CD8 T-cell line hCD8-NLV were stained for the expression of CD28 and CD95/Fas to identify CD28+CD95low TN, CD28+CD95high TCM, and CD28-CD95high TEM subsets. Further characterization of the phenotype of hCD8-NLV cells included the T-cell differentiation markers CD45RA, CD45RO, CD62L, and CCR7. (TIF) [file ppat.1005049.s001.tif]

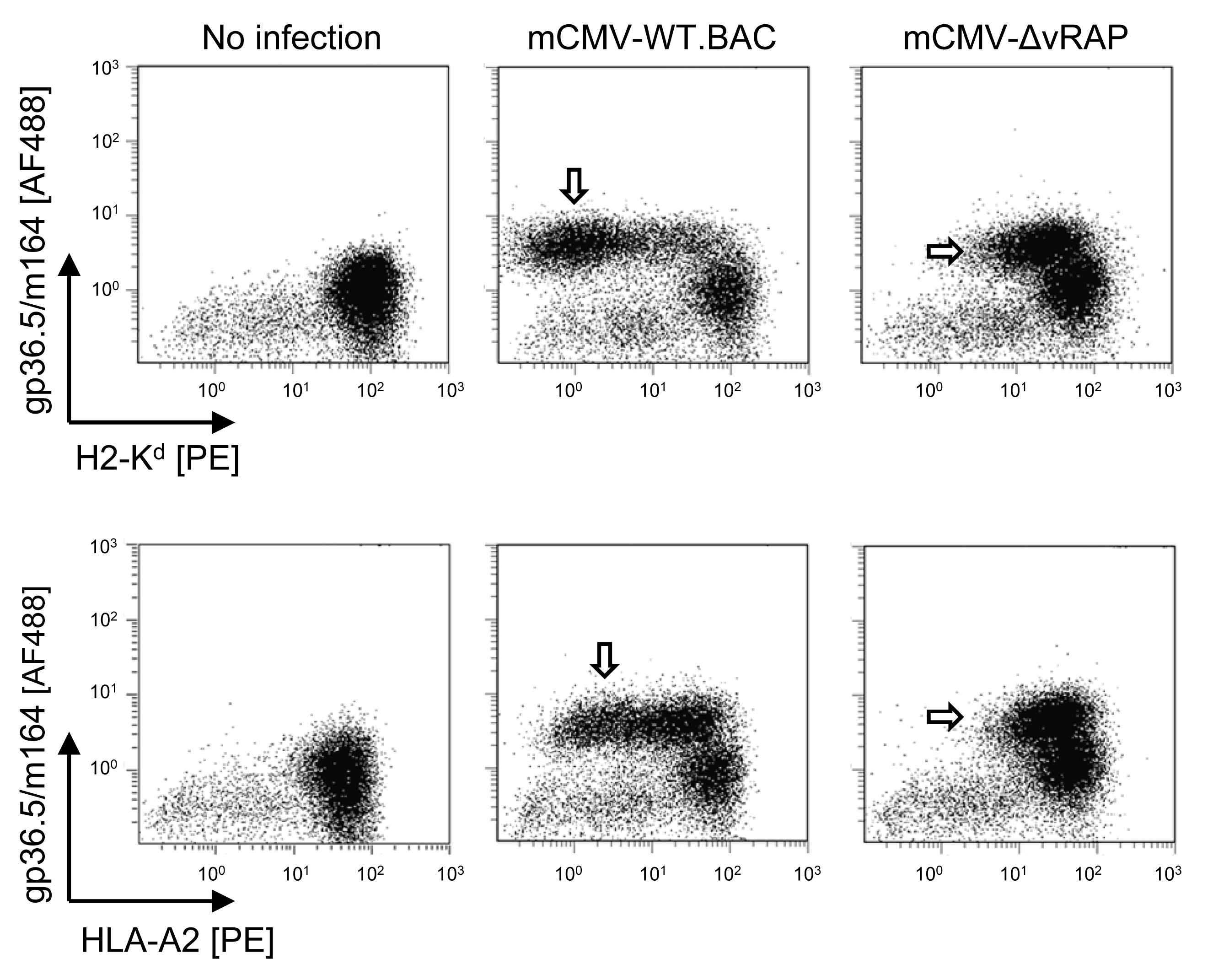

Supplement: S2 Fig — MEF derived from NSG/HHD mice were pre-treated with IFN-γ for 48h and infected with the indicated viruses. Shown are cytofluorometric 2D dot plots of cell surface MHC-I expression (H2-Kd, upper panel; HLA-A2.1, lower panel; abscissa: PE-fluorescence intensity) depending on the infection of cells indicated by expression of the intracellular infection marker gp36.5/m164 (ordinate; Alexa Fluor488-fluorescence intensity). Arrows point to the infected gp34/m164+ cell population that is MHC-Ilow after infection with mCMV-WT.BAC and MHC-Ihigh after infection with mCMV-ΔvRAP, in which genes encoding the viral regulators of antigen presentation (vRAP) gp34/m04, gp48/m06, and gp40/m152 are deleted. Data are representative of two independent experiments. (TIF) [file ppat.1005049.s002.tif]

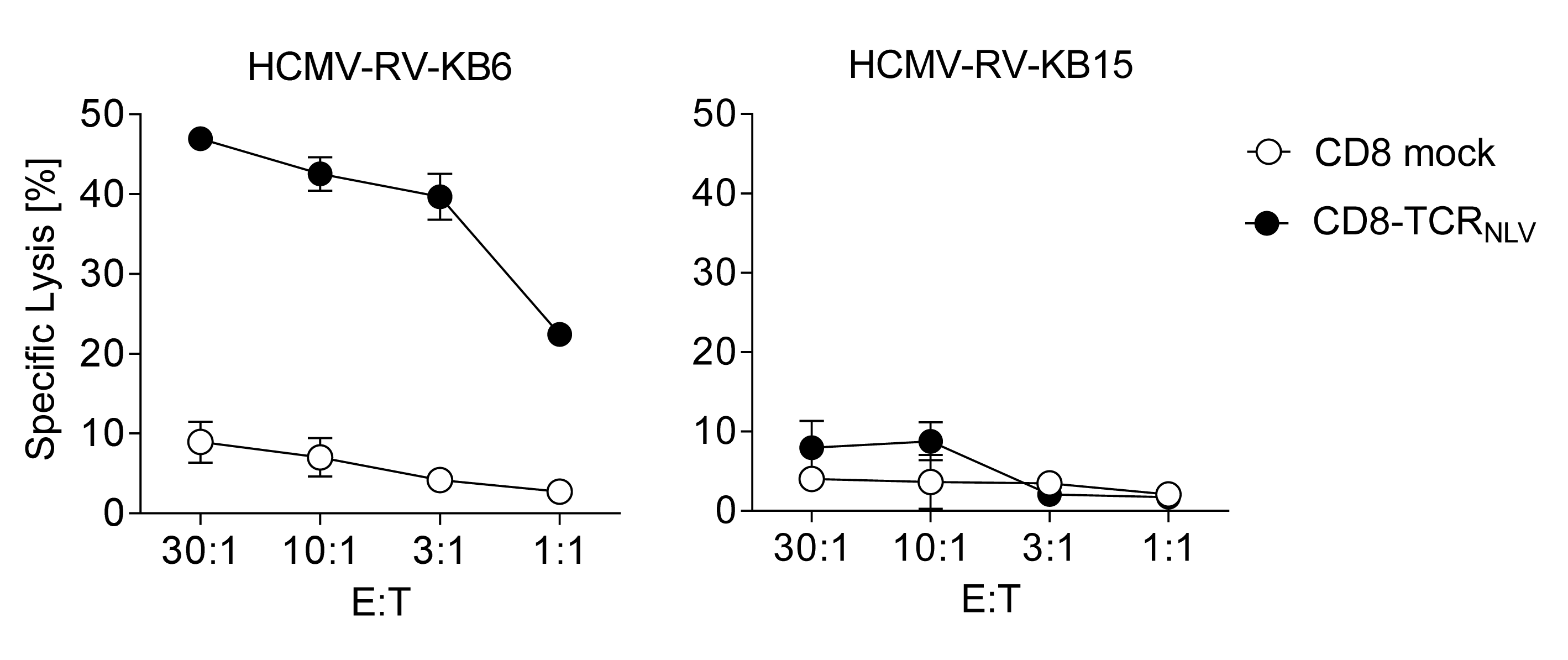

Supplement: S3 Fig — Immunomagnetically-selected human CD8 T cells were retrovirally transduced with TCRNLV (CD8-TCRNLV, filled circles) or empty vector (CD8 mock, open circles). After in vitro expansion with anti-CD3/CD28 beads for a period of 10d, cells were analyzed at the indicated effector-to-target (E:T) cell ratios for cytolysis of HLA-A2.1+ human primary foreskin fibroblasts infected with HCMV immune evasion gene deletion mutant RVKB6 (NLV+) or with the combined immune evasion and pp65/UL83 deletion mutant RVKB15 (NLV-). Data represent means of duplicate assay cultures. Error bars indicate the range. (TIF) [file ppat.1005049.s003.tif]

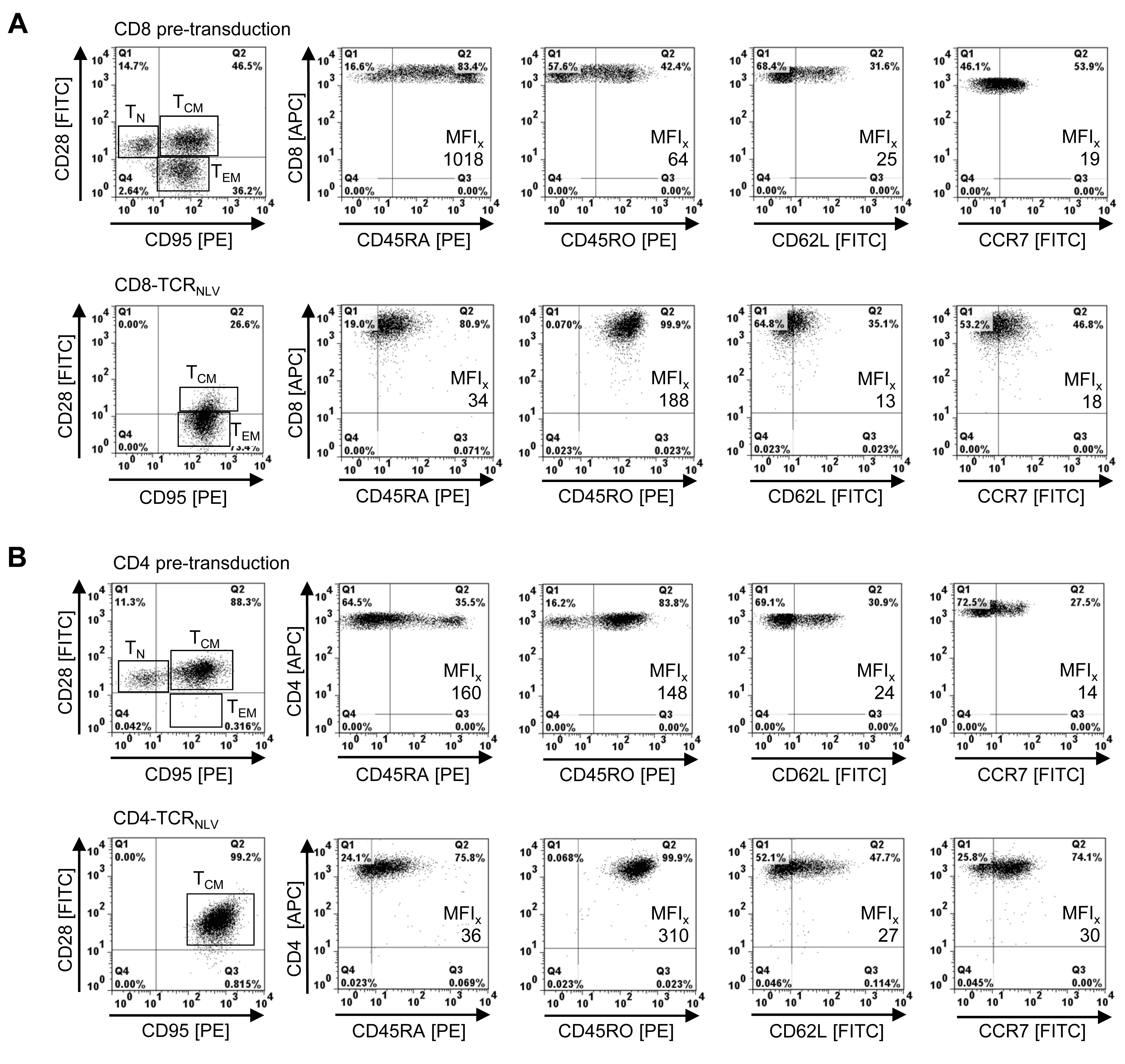

Supplement: S4 Fig — Immunomagnetically selected CD8 (A) and CD4 (B) T cells were stained for cytofluorometric phenotyping before (upper panels) and after retroviral transduction with TCRNLV (CD8-TCRNLV and CD4-TCRNLV cells, respectively) followed by in vitro expansion for a period of 10d (lower panels). Cell surface markers CD28 and CD95/Fas identify CD28+CD95low naive (TN), CD28+CD95high central memory (TCM), and CD28+CD95high effector-memory (TEM) T cells. Further characterization of the phenotype included the T-cell differentiation markers CD45RA, CD45RO, CD62L, and CCR7. Shown are 2D dot plots. Percentages and mean-fluorescence intensities (MFI) of labeled cells are indicated. (TIF) [file ppat.1005049.s004.tif]

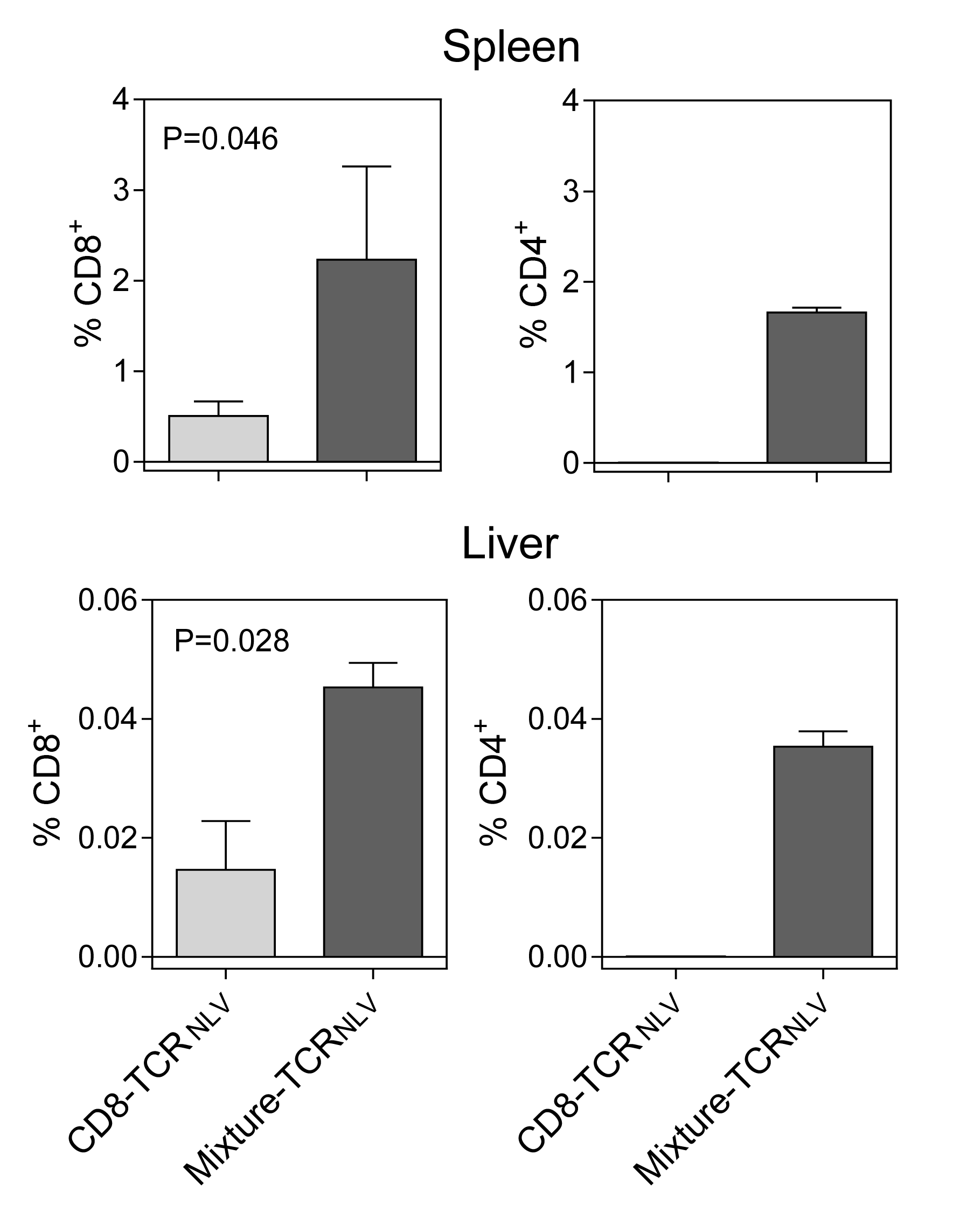

Supplement: S5 Fig — Corresponding to data on the control of organ infection (see the legend of Fig 6), adoptively transferred CD8-TCRNLV cells (left panels) and CD4-TCRNLV cells (right panels) were analytically retrieved by cytofluorometric analysis from spleen (upper panels) and liver (lower panels) of NSG/HHD mice on day 3 after intraplantar infection with 1x10^5^ PFU of mCMV-NLV. Grey-shaded bars: retrieval after transfer of 1x10^7^ CD8-TCRNLV cells. Black bars: retrieval after transfer of a mixture consisting of 2x10^6^ CD4-TCRNLV and 8x10^6^ CD8-TCRNLV cells. Bars represent mean % values of data from three individual mice. Error bars indicate SEM. P values for significance of differences were calculated by using the ratio paired t-test. (TIF) [file ppat.1005049.s005.tif]

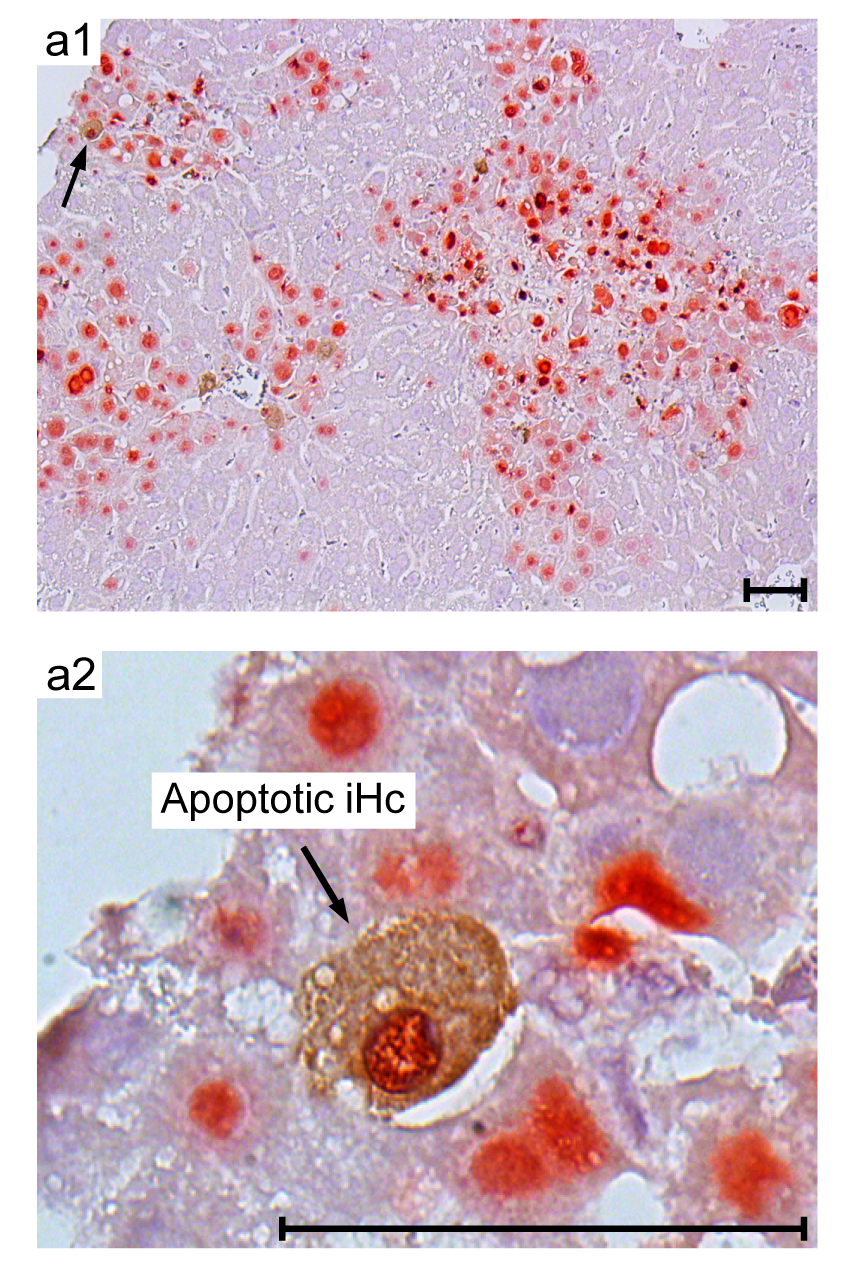

Supplement: S6 Fig — Corresponding to the 2C-IHC analysis of liver tissue infection and apoptosis shown in Fig 10, where uninfected, apoptotic hepatocytes (Hc) were found to be located in foci of infection, images here show an example of an infected, apoptotic hepatocyte (iHc) identified by co-expression of intranuclear IE protein (red staining) and cytoplasmic active caspase 3 (brown staining). (a1) overview of liver tissue infection; the arrow points to a region that is resolved to greater detail in the higher magnification image (a2). Bar markers: 50 μm. (TIF) [file ppat.1005049.s006.tif]
